# Competitive fitness of *Pseudomonas aeruginosa* isolates in human and murine precision-cut lung slices

**DOI:** 10.3389/fcimb.2022.992214

**Published:** 2022-08-23

**Authors:** Nina Cramer, Marie Luise Nawrot, Lion Wege, Marie Dorda, Charline Sommer, Olga Danov, Sabine Wronski, Armin Braun, Danny Jonigk, Sebastian Fischer, Antje Munder, Burkhard Tümmler

**Affiliations:** ^1^ Clinical Research Group ‘Pseudomonas Genomics’, Department for Pediatric Pneumology, Allergology and Neonatology, Hannover Medical School, Hannover, Germany; ^2^ Biomedical Research in Endstage and Obstructive Lung Disease Hannover (BREATH), German Center for Lung Research, Hannover Medical School, Hannover, Germany; ^3^ German Center for Infection Research, Hannover Medical School, Hannover, Germany; ^4^ Research Core Unit Genomics, Hannover Medical School, Hannover, Germany; ^5^ Fraunhofer Institute for Toxicology and Experimental Medicine (ITEM), Member of Fraunhofer International Consortium for Anti-Infective Research (iCAIR), Hannover, Germany; ^6^ Institute of Pathology, Hannover Medical School, Hannover, Germany

**Keywords:** chronic bacterial infection, cystic fibrosis, *pseudomonas aeruginosa*, adaptation, host defense, precision-cut lung slices, *ex vivo* model

## Abstract

Chronic respiratory infections with the gram-negative bacterium *Pseudomonas aeruginosa* are an important co-morbidity for the quality of life and prognosis of people with cystic fibrosis (CF). Such long-term colonization, sometimes lasting up to several decades, represents a unique opportunity to investigate pathogen adaptation processes to the host. Our studies aimed to resolve if and to what extent the bacterial adaptation to the CF airways influences the fitness of the pathogen to grow and to persist in the lungs. Marker-free competitive fitness experiments of serial *P. aeruginosa* isolates differentiated by strain-specific SNPs, were performed with murine and human precision cut lung slices (PCLS). Serial *P. aeruginosa* isolates were selected from six mild and six severe CF patient courses, respectively. MPCLS or hPCLS were inoculated with a mixture of equal numbers of the serial isolates of one course. The temporal change of the composition of the bacterial community during competitive growth was quantified by multi-marker amplicon sequencing. Both *ex vivo* models displayed a strong separation of fitness traits between mild and severe courses. Whereas the earlier isolates dominated the competition in the severe courses, intermediate and late isolates commonly won the competition in the mild courses. The status of the CF lung disease rather than the bacterial genotype drives the adaptation of *P. aeruginosa* during chronic CF lung infection. This implies that the disease status of the lung habitat governed the adaptation of *P. aeruginosa* more strongly than the underlying bacterial clone-type and its genetic repertoire.

## Introduction

Cystic fibrosis (CF) is one of the most common severe monogenic traits in populations of European descent (Bell et al., 2020; Shteinberg et al., 2021). Thanks to the continuous improvement of the symptomatic treatment programs (Lopes-Pacheco, 2020; Konstan et al., 2021: Schlüter et al., 2021) and the recent development of highly efficient CFTR modulator therapies (HEMT) (Heijerman et al., 2019; Middleton et al., 2019; Barry et al., 2021; Zemanick et al., 2021) the quality of life and prognosis for CF patients have meanwhile substantively increased. However, the chronic airway colonization with opportunistic pathogens, particularly *Staphylococcus aureus* and *Pseudomonas aeruginosa*, are rather refractory to therapeutic intervention (Muhlebach et al., 2021; Durfey et al., 2021) and will remain the major co-morbidity in people with CF for many years to come, albeit HEMT has recently been demonstrated to substantially reduce the bacterial load with these opportunistic pathogens (S.T. Pallenberg, personal communication).

*P. aeruginosa* is the most prevalent pathogen in the airways of CF adolescents and adults (Parkins et al., 2018; Blanchard and Waters, 2019). Once *P. aeruginosa* has successfully conquered the patients’ airways, the bacterium will persist and adapt to its niches in the upper and lower respiratory tract (Camus et al., 2021). The microevolution of *P. aeruginosa* in the CF airways (Winstanley et al., 2016) has been investigated by whole genome sequencing of isolates from unique patients (Cramer et al., 2011; Smith et al., 2016) and major transmissible lineages (Marvig et al., 2013; Moore et al., 2021) and finally of serial isolates from CF patient cohorts (Marvig et al., 2015; Klockgether et al., 2018).

The *P. aeruginosa* strain collection from the Hannover CF clinic is a unique source of serial CF airway isolates that have been collected in regular intervals from 35 patients since the onset of colonization in the 1980s until today (Cramer et al., 2012; Klockgether et al., 2018; see **Supplementary Table S1**). We selected the six mildest and most severe patients’ courses of the *P. aeruginosa* infection, respectively, for in depth genomic and phenotypic characterization of their serial clonal *P. aeruginosa* isolates (Klockgether et al., 2018). Our previous studies showed the influence of adaptation to the clones’ fitness by subjecting an early isolate and its clonal descendants to competitive growth *in vitro* (Cramer et al., 2020). To better cope with the natural conditions of the airways, we here use murine and human precision cut lung slices (PCLS) to investigate whether the microevolution influenced the bacterial fitness to grow in the presence of lung tissue. PCLS are an organotypic *ex vivo* model that retain the natural heterogeneity of lung physiology and morphology with all cell types present (Switalla et al., 2010; Neuhaus et al., 2018). The outcome of the competitive fitness growth experiments was substantially different between the two groups with mild or fatal chronic infections. Whereas early isolates dominated the competition in clonal lineages isolated from severely affected CF patients, intermediate and/or late infection isolates commonly won the competition in clonal lineages from mildly infected patients.

## Material and methods

### Strain collection

*P. aeruginosa* strains were isolated from 35 CF patients who were regularly attending the CF clinic at Hannover Medical School and who became chronically colonized in their airways with *P. aeruginosa* between 1984 to 1992 (Cramer et al., 2012). *P. aeruginosa* isolates collected in half-year intervals were stored in duplicates as glycerol stock cultures at - 80°C, giving rise to a collection of sequential isolates over a period of up to 40 years.

Since 2010 the clone type of isolates has been determined with a multi-marker microarray (Wiehlmann et al., 2007). About 500 sequential descendants were sequenced of the clonal lineages of the six mildest and the six most severe patient courses (Klockgether et al., 2018). The classification whether the course was assessed as severe or mild, was based on the archived Body Mass Index and the FEV1 spirometry values of patients. All isolates (as long as the original detected clone was maintained) of the 12 longitudinal courses were checked for unique strain-specific SNPs. In total, 129 bacterial strains distinguishable *via* SNPs were found to be suitable for a marker-free isolate identification. A SNP-spanning multiplex PCR with subsequent amplicon sequencing was established and tested in competitive fitness experiments *in vitro* (Cramer et al., 2020).

After optimization of the amplification of the individual strain-specific primer pairs to one uniform multiplex PCR condition, 98 of these 129 strains passed this quality control of a clearly visible gel-separated PCR product and hence were selected for the *ex vivo* fitness experiments in PCLS (see **Supplementary Table S1**). Conditions for the Multiplex PCR are described in chapter 2.6.

### Culture media

Bacteria were grown in either liquid Lysogeny broth (LB) for 6h/12h at 37°C and 150 rpm or on solid LB agar plates overnight at 37°C.

### Precision- cut lung slices

To mimic real-life lung tissue environment as close as possible, competitive fitness experiments were performed in murine and human PCLS (Switalla et al., 2010; Neuhaus et al., 2018).

#### Mouse precision-cut lung slices

Murine PCLS were prepared from lungs of 8-10 week old mice of strain C57BL/6J (obtained from the Central Animal Facility of the Hannover Medical School). Mice were sacrificed by an overdosed intraperitoneal anaesthesia (6.67 mg ketamine + 0.67 mg xylazine per 10 g body weight). When breathing stopped, the diaphragma and the thorax were opened and the lungs were filled with 1.5% (w/v) low-gelling agarose (A4018, Sigma-Aldrich) through a cannula in the trachea until all lung lobes were completely expanded. After polymerization of the agarose, the whole heart lung package was removed and stored in phosphate buffered saline (PBS) on ice until sectioning. Mouse lungs were dissected into single lobes, and lobes were cut with an automatic oscillating microtome (7000smz-2, Campden Instruments, Loughborough, England) in ice-cold Earle´s balanced salt solution (EBSS) into 320 µm thin slices. After three washing steps with Dulbecco´s Modified Eagle Medium with Ham´s F-12 (DMEM/F-12 medium) at 37°C, 5% CO_2_, two slices each were transferred into a well of a 24-well plate, filled with 500 µl DMEM/F-12 medium and cultured at 37 C and 5% CO_2_ until infection. It was demonstrated in previous studies that PCLS, prepared according to this protocol, show reactions of living tissue such as vasoconstriction, bronchoconstriction or the release of cytokines towards infection stimuli during at least the first 48h (Henjakovic et al., 2008; Danov et al., 2019).

#### Human precision-cut lung slices

Human PCLS (patient’s age ranged from 34 to 76 years; 7 male and 5 female) were obtained from histologically unremarkable border areas of resected lung tumor tissue or from non-CF end-stage lung explants (ethic approval number 2701-2015; **Supplementary Table S1**, third tab). Tissue of CF lungs was excluded *a priori* due to profound tissue damage and massive bacterial load, which are characteristics for end-stage CF lungs.

To obtain hPCLS, lungs were filled with 2% (w/v) low-gelling agarose. After polymerization on ice, lung tissue was cut into slabs, and cylindrical biopsies with 8 mm diameter were punched out. PCLS slices were cut with approximately 300 µm in width in ice-cold EBSS using a Krumdieck tissue slicer (Alabama Research and Development, Muniford, AL, USA). After three washing steps with DMEM/F-12 at 37°C, 5% CO_2_, two slices each were transferred into a well of a 24-well plate, which was filled with DMEM/F-12 and cultured like the mPCLS at 37°C and 5% CO_2_ until infection (not longer than 12 h). Biological replicates each executed in triplicate were performed with tissue from the same lung and anatomic region.

### Infection protocol

**Figure 1:**
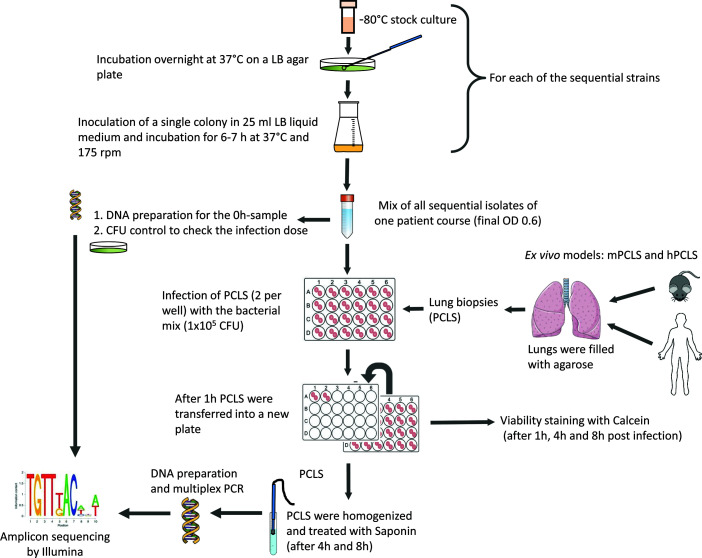
Outline of the competitive fitness experiments in murine and human PCLS.

For infection, all serial isolates of a course were grown separately in LB-medium overnight at 37°C and inoculated into fresh LB medium 6-8 h before infection in order to ensure that the bacteria were in an exponential growth phase at the beginning of the competition experiment. **Figure 1** illustrates the protocol of a competitive fitness experiment. Based on measurements of the optical density, the serial isolates from early exponential phase were mixed equally (total OD 0.6), and subsequently mPCLS or hPCLS were infected with 50 µl of this bacterial mixture in triplicate (final bacterial concentration 1x10^5^ CFU determined to be optimal for harvesting sufficient amounts of bacterial DNA for multiplex PCR in a large background of mammalian DNA without compromising PCLS vitality until the endpoint). The linear correlation between CFU and OD measurements during early exponential phase had been validated in preliminary experiments (Cramer et al., 2020). Prior to inoculation, CFUs of the mixture were assessed by plating on LB agar plates, whereby one aliquot was used for the CFU determination and another aliquot for DNA preparation and subsequent sequencing. The infected slices were incubated in the 24-well plates for one hour at 37°C and 5% CO_2_ and then transferred into new wells to ensure that only bacteria adhering to the PCLS or those which already had invaded the cells had an influence on the further course of the experiment.

Samples for sequencing were collected 4 h and 8 h after infection. The PCLS were transferred into 500 μl fresh PBS, homogenized and lysed with saponine to get access to the endocytosed bacteria. After centrifugation at 3800 g for 8 min, the supernatant was discarded and the bacterial DNA was isolated from the cell pellet by phenol-chloroform extraction (Dale and Greenaway, 1985).

### Calcein staining

Viability of the PCLS of technical replicates and negative controls at time points 1 h, 4 h, and 8 h was determined by staining with calcein. After removal of medium from the slices, 500 μl of 4 mM calcein acetoxymethyl (AM) solution (ThermoFisher) was added per well. As a dead control two slices were pre-incubated with 500 μl of 70% ethanol for 30 min.

After 45 minutes of incubation in the dark at 37°C and 150 rpm, the supernatant was removed by washing three times with 500 μl of DMEM/F-12. The PCLS were then lysed by adding 500 μl of 1% (v/v) Triton-X100 solution per well and incubating for 30 min at 4 C. The supernatants of the three technical replicates were combined and mixed for at least 20 s. Fluorescence was measured in duplicate at 485/517 nm (excitation/emission wavelength) with a band width of 20 nm.

### Multiplex PCR

In order to enable competitive fitness experiments of various serial *P. aeruginosa* isolates, we have developed a multiplex marker-free amplicon pipeline (Cramer et al., 2020). For this purpose, sequenced serial isolates were examined for strain-specific SNPs. Based on this information, quantitative differentiation of serial isolates became possible without the need of any additional external marker such as fluorescent conjugates. One to four specific SNPs per isolate were selected from in total 98 isolates, i.e. 240 and 236 SNPs for mPCLS and hPCLS experiments, respectively. SNP-spanning PCRs were optimized with an approximate target length of 200 bp. To minimize time and costs, conditions for multiplex PCRs of genomic DNA extracted from infected PCLS with up to ten primer pairs were established. Accordingly, the following optimized protocol was used for the amplification of *P. aeruginosa* DNA in the background of mammalian DNA:

Multiplex-amplicon-PCR was performed with 35 cycles (60 s at 60°C, 60 s at 72°C and 90 s at 94°C) with 50 ng DNA, 15 pmol per primer, 2 mM MgCl_2_, 2% DMSO (v/v) and 1 unit Goldstar polymerase (Eurogentec, Liège, Belgium). Primer pairs spanning a strain-specific SNP were designed by web-based ‘Primer 3 Input’ (http://bioinfo.ut.ee/primer3) for one to four SNPs per isolate.

### Fragment libraries/amplicon sequencing

Multiplex PCR products of single technical replicates of 2 x 6 patient courses from the same time point and habitat (mPCLS or hPCLS) were combined in 152 samples for library preparation and sequencing. To achieve a sufficiently high yield of amplicon reads and to avoid any overlaps of amplicon sequences caused by vicinal strain-specific SNPs, only amplicons of six courses were combined.

100 - 250 ng of DNA per amplicon were utilized as input for preparing fragment libraries with NEBNext^®^ Ultra II DNA Library Prep Kit for Illumina^®^ (E7645L; New England Biolabs). All steps were performed as recommended in the user manual E7645 (Version 5.0_06-2018; New England Biolabs) except that all reaction volumes were downscaled to 1/2 of the recommended volumes. DNA libraries were barcoded by unique dual indexes (8bp), using NEBNext^®^ Multiplex Oligos for Illumina^®^ (96 Unique Dual Index Primer Pairs, E6440S, New England Biolabs).

All generated DNA libraries were amplified by six to eight PCR cycles. DNA concentration was quantified by using the ‘Qubit^®^ dsDNA HS Assay Kit’ (Q32854; ThermoFisher Scientific). After pooling equimolar amounts, denaturation and dilution to 10 pM (MiSeq) or 1.8 pM (NextSeq), sequencing was performed on an Illumina^®^ MiSeq- System for mPCLS or a NextSeq 550 platform for hPCLS competition experiments.

### Processing of sequence data

Of the Illumina MiSeq and NextSeq paired-end reads the first 20 bases and all bases after position 150 were trimmed with the program fastx_trimmer (http://hannonlab.cshl.edu/fastx_toolkit/index.html). Paired reads were merged and aligned to the reference genome PA14 (Lee et al., 2006) using ‘bwa aln’ (Li and Durbin, 2009). The number of reads carrying strain-specific SNPs was extracted directly from the generated sam-files using an in-house script (https://github.com/kfgpfz/position_extraction).

Next, the sum of all isolates was normalised to 100% within the longitudinal course. The relative changes from 0 h - 4 h and from 0 h - 8 h and the mean percentage of the three technical replicates (± 2 σ) were calculated (if at least two of the three technical replicates showed results) as previously described in Cramer et al., 2020.

**Figure 2:**
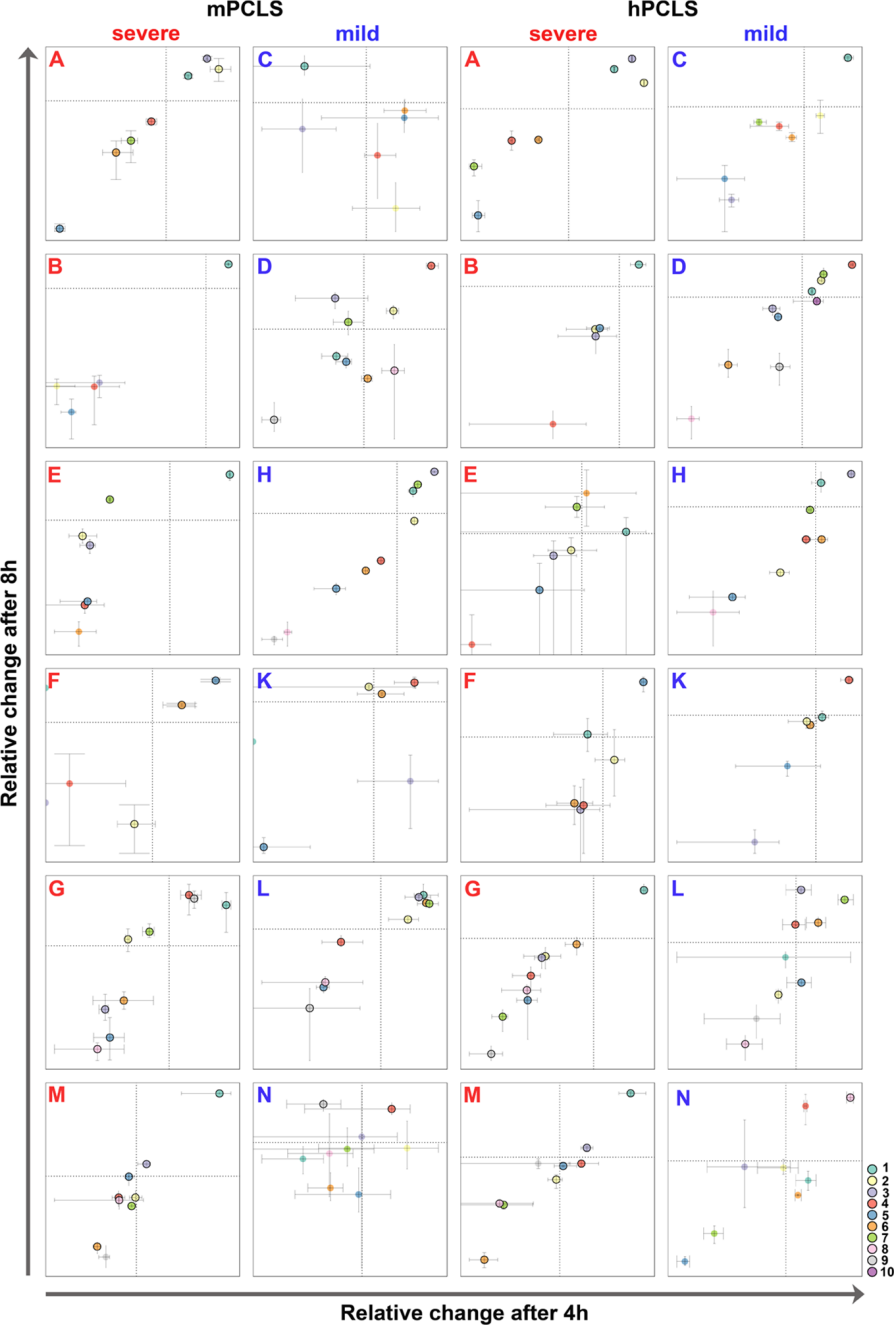
Outcome of competitive fitness experiments of 12 longitudinal courses in murine (left) and human PCLS (right). Relative growth changes of serial *P. aeruginosa* isolates during competitive fitness experiments are compared at 4 h and 8 h, respectively. Severe longitudinal courses are labeled with red, mild courses with blue letters. Each serial isolate is differentiated by color sorted by increasing colonization time of the clone in the patient’s lungs: *P. aeruginosa* strains marked without a black outer ring were recovered at such low quantities that the strain-specific SNP counts were classified as ‘detectable, but not quantifiable’ (see Material and methods section). The axis labeling as well as the scaling and the color of the isolates are explained in more detail in **Supplementary Dataset S1** showing all plots at higher resolution. **Supplementary Figure S1** shows the outcome of the other biological replicate.

The relative quantity of a strain at 4 h and 8 h compared to 0 h was visualized by the mean and range of the three replicates in a two-dimensional plot. Outliers were eliminated if individual values of strain-specific SNPs exceeded the range of mean ± 2 σ. If the mean number of reads assigned to a strain was below 0.5% of the amplicon reads in at least two of three technical replicates, the bacterial strain was classified as ‘detectable, but not quantifiable’ (marked without a black line in **Figure 2**).

### Statistical analysis

The Kruskal-Wallis test was used to check the scatter within the technical triplicates (**Supplementary Table S3**). The Wilcoxon signed rank test was used for comparison of biological replicates (**Supplementary Table S3**).

All values of a course were examined by both the Kolmogorov-Smirnov test and ANOVA whether they were normally distributed. Variance homogeneity was checked by Levene´s test (**Supplementary Table S2**). If data were not normally distributed, non-parametric tests (Kruskal-Wallis and/or Wilcoxon) were applied.

## Results

We performed competitive fitness experiments with six to ten serial *P. aeruginosa* serial clonal isolates collected from the same CF patient since the onset of colonization until fatal outcome (six severe courses A, B, E, F, G, M) or up to the last isolate prior to clone replacement ten to thirty years later (six mild courses C, D, H, K, L, N) (21) (**Supplementary Table S1**). Each of these 12 patients was chronically harboring a genetically distinct *P. aeruginosa* clone. Individual isolates of these longitudinal courses were differentiated by strain-specific single nucleotide variants (SNPs) in locus-specific amplicon reads generated by deep multiplex sequencing (Klockgether et al., 2018). We have shown in a previous study that singular strains from sets of these serial clonal isolates showed indistinguishable growth characteristics in liquid culture (Cramer et al., 2020). Within-clone competition was performed for eight hours in PCLS generated from lungs of mice (C57BL/6J) or from histo-morphologically normal human tissue of the resection margin from lung tumors (hPCLS) (**Figure 1**; additional information about donors of the hPCLS see **Supplementary Table S1**). Competition experiments longer than 8 hours were not feasible due to massive bacterial growth and decrease of vitality of the infected tissue. Since PCLS showed untarnished viability in the Calcein-staining during all competitive experiments after 8h, this time point was selected as endpoint for the analysis.

### Competition in mPCLS

**Figure 3:**
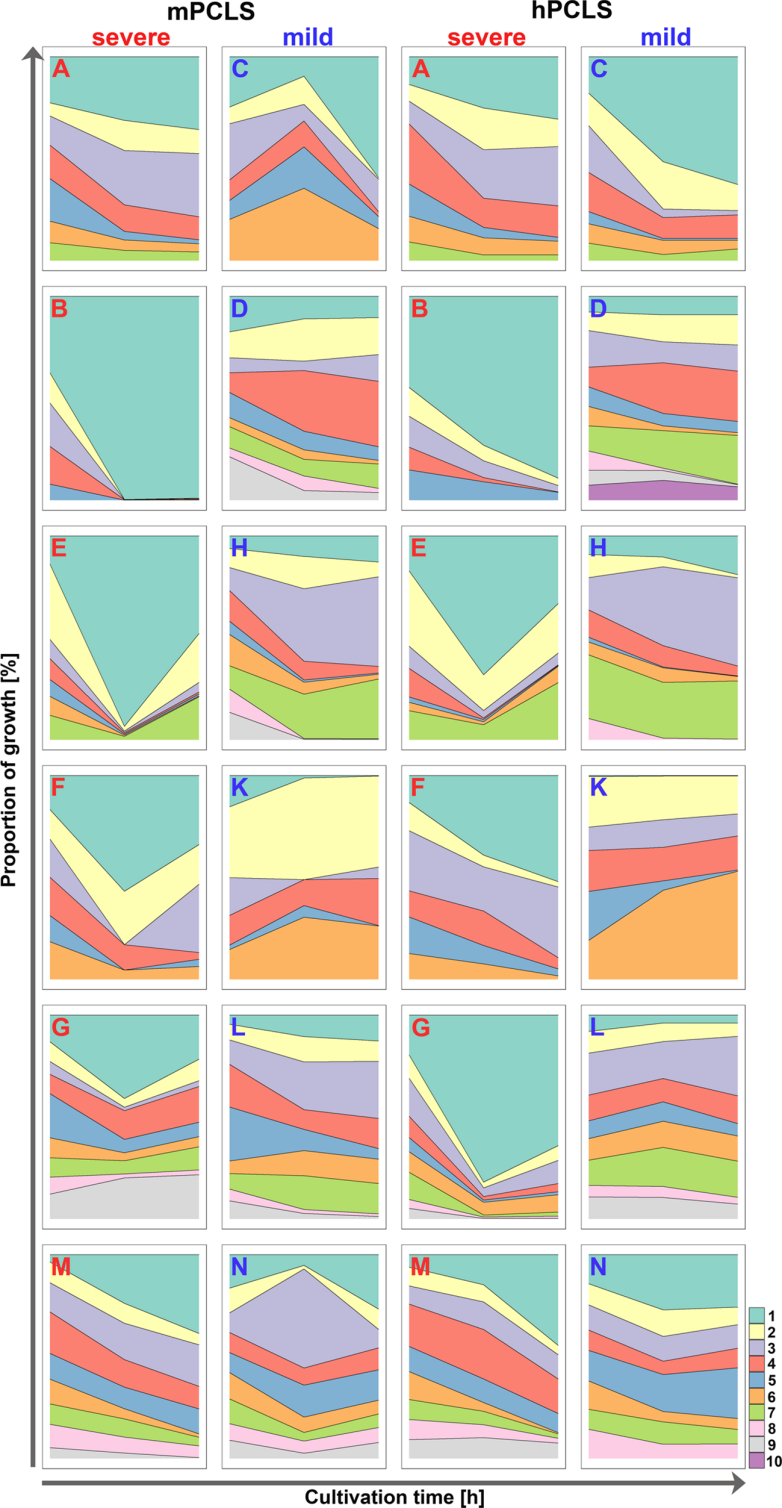
Relative proportions of serial isolates during competitive growth in murine (left) and human PCLS (right) after 4 hours and 8 hours. For each serial isolate of the twelve longitudinal courses, its fraction in the clonal community at the time points 0 h, 4 h and 8 h is shown. Longitudinal courses are differentiated by a bold letter (severe courses in red; mild courses in blue). Each serial isolate is depicted by color sorted by increasing colonization time of the clone in the patient’s lungs. For more detailed information see **Supplementary Dataset S1**. For outcome of the second biological replicate see **Supplementary Figure S2**.

**Figure 4:**
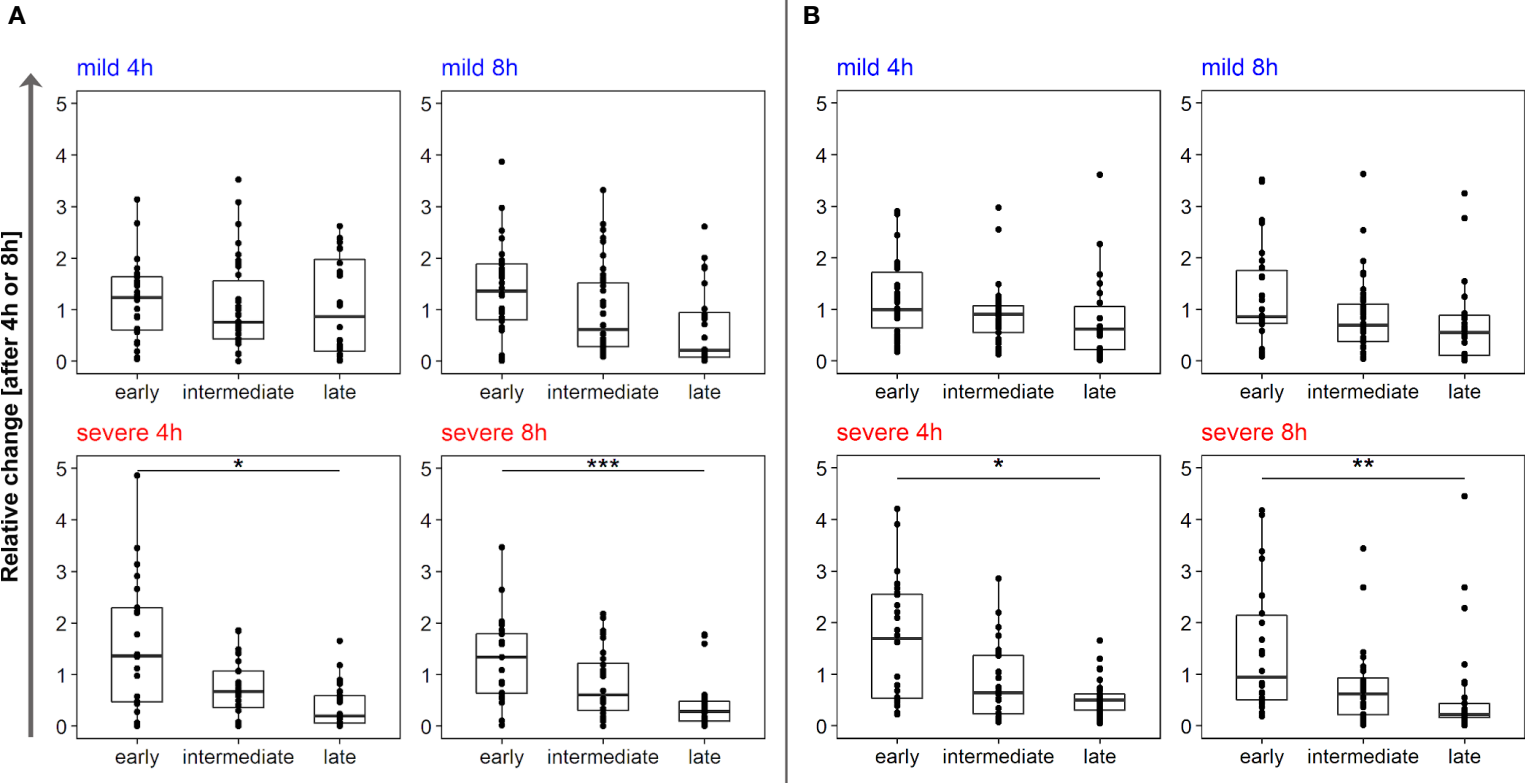
Outcome of competitive fitness experiments in mPCLS and hPCLS after 4 h and 8 h. Serial *P. aeruginosa* isolates from mild courses (upper panel) and severe courses (lower panel) were grouped by colonization time in CF patients’ lungs time in early, intermediate and late isolates (see **Supplementary Tables S4**/**S5** for details). Relative growth changes are shown for the individual strains of the three groups. The number of data points per group depends on the number of isolates assigned to that group. Since early/late isolates were assigned to the first/last quartile of the infection period, respectively, the intermediate isolates extend to the inner two quartiles and are therefore quantitatively superior. Statistics and thus the significance were calculated with the help of Kruskal-Wallis. All *p-*values were corrected for multiple testing by Bonferroni correction.

In general, competition experiments in murine PCLS (mPCLS) revealed that only a minority of isolates was actively growing while the majority of the clonal variants grew poorly (all raw data/quantities of each serial isolate are available at https://github.com/NinaCramer/results-competitive-fitness-mPCLS-hPCLS.git). Even though the same CFU of each clonal variant had initially been inoculated, the numerical distribution of strains did not follow a narrow Gaussian distribution after 4 or 8 hours of growth in the presence of clonal competitors (**Supplementary Table S2**), whereas CFU of biological and technical replicates were comparable (**Supplementary Table S3**). The growth rates of the individual strains and their relative proportions within the bacterial community were estimated at time points 4 h and 8 h from the start of the experiment (**Figures 2**, **3**; biological replicates see **Supplementary Figure S1**). For detailed description of graphical presentations see **Supplementary Dataset S1**. Depending on the individual patient’s course, one (courses B, C, E, M, N), two (D, F, K), three (A, G, H) or four strains (L) displayed active growing (**Figures 2** and **3**, column 1 and 2). In all but the two mild courses D and K (*P_4h_* = 0.05; *P_8h_* = 3.2 x 10^-5^, Kruskal-Wallis), the “winner” strains were represented by the first two (4 h) or four (8 h) serial isolates. The initial isolate retrieved close to the onset of chronic colonization of the patient’s lungs was the dominant winner of the competition in severe (*P* < 0.0001), but not mild courses of infection (*P* = n.s) (**Supplementary Table S3**). Next, the strains were separated into three groups depending on colonization time (early isolates = first quartile of colonization, intermediate = the two inner quartiles of the colonization time; late isolates = last quartile of colonization time) (**Supplementary Table S4**). Early isolates from severe, but not from mild courses of infection grew significantly better than the late isolates (early vs. late severe: *P_4h_* = 0.9 x 10^-2^, *P_8h_ =* 5.0 x 10^-4^) (**Figure 4**, **Supplementary Table S3**). According to this data, *P. aeruginosa* isolates from mild chronic CF lung infections were growing equally well in murine PCLS derived from healthy murine lungs irrespective of their temporal occurrence in the patient’s lung. In contrast, early isolates of the severe courses were growing significantly better than their later clonal progeny.

### Competition in hPCLS

We performed analogous competitive fitness experiments in PCLS freshly prepared from explanted human lung tissue (raw data and quantities see link above), an *ex vivo* habitat very closely resembling real-world conditions than murine lung slices. Both age and gender of lung tissue donors (**Supplementary Table S1**) were not significantly different between the subgroups selected for isolates from severe and mild clinical courses. The results obtained with mPCLS and hPCLS are shown side-by-side in **Figures 2** and **3** (in more detail see **Supplementary Dataset S1**). Consistent with the mPCLS model, the early *P. aeruginosa* isolates emerged as winners in the hPCLS competition experiments if clones had been retrieved from patients with severe CF courses. Conversely, no unequivocal trend was observed with the clonal strain collections from patients who experienced mild disease courses over decades of chronic *P. aeruginosa* airway colonization. Of the 12 courses, one (course B, C, F, G, K), two (A, E, M, N) or four isolates (D, H, L) showed a positive growth rate over the experimental time. Most winner strains belonged to the first four isolates (*P* < 0.0001), whereby the initial isolate collected at the onset of airway colonization was the absolute winner in seven (4 h) or six courses (8 h) (*P* < 10^-5^) and showed the highest growth rate in all severe courses (*P* < 0.0001) (**Supplementary Table S3**). If the isolates were grouped by colonization time (see above), the panel of early isolates of the severe courses showed a significantly higher growth rate than that of the late isolates (*P_4h_* = 0.05; *P_8h_* = 1.6 x 10^-3^; **Supplementary Table S3**).

## Discussion

With the start of CFTR modulator therapy, the therapeutic options for people with CF have improved immensely and life expectancy increased to an average of up to 50 years (Bell et al., 2020), but 44% of European CF patients are still chronically colonized with *P. aeruginosa* (Orenti et al., 2021). The *CFTR* mutation genotype, disease pathophysiology and gender-specific factors (Di Paola et al., 2017) modulate CF airways and thus the susceptibility of the niche to be conquered by opportunistic pathogens such as *P. aeruginosa*. To investigate the influence of *de novo* mutations in the *P. aeruginosa* genome acquired during CF lung colonization on the fitness of a strain, we set up competitive fitness experiments with serial isolates from mild and severe courses of infection in the *ex vivo* PCLS lung model. Up to 10 serial isolates of the same clone, which span the entire course of infection, were grown together in PCLS.

Our fitness experiments in both the murine and the human *ex vivo* lung slices consistently revealed a distinct outcome of the competition experiments depending on the clinical course of CF patients. Bacterial strains collected from the early phase of lung colonization in CF patients with progressively severe courses became dominant strains with high frequency in the applied *ex vivo* models. This growth advantage was not observed in early isolates from CF patients with persistently mild disease courses. Here, mid-term or late isolates were found more frequently among the winner strains (**Figures 2**–**4**). According to reports from our colleagues (reviewed by Camus et al., 2021) *P. aeruginosa* rapidly diversifies during the initial adaptation phase to the CF lung (which represents the first 4 years of colonization in our cohort) followed by a consolidation phase with less changes in genotype and phenotype. These findings imply that this initial positive selection may favor the early isolates to win the competition whereas the subsequent neutral or even negative selection will cause a loss of competitive fitness of the clonal descendants.

Previously reported *in vitro* experiments observed an increased fitness of single strains with rising mutation rates of *P. aeruginosa* (Grekov et al., 2020). However, in our context of severe CF lung infections the early isolates, which had not acquired many pathoadaptive mutations, had a higher competitive growth fitness in PCLS than their clonal descendants. Early *P. aeruginosa* isolates won the competition irrespectively of whether they had been collected from mild or severe courses. In other words, early isolates unanimously were the “winners” of the competition, when cultivated under standardized growth conditions. This imbalance towards the isolates from the onset of colonization was most pronounced in a fermenter where growth conditions were under rigorous external control. It became less prominent in the *ex vivo* murine habitat and least apparent in the human *ex vivo* tissue closest to real-life conditions.

The genetic adaptation of *P. aeruginosa* to CF airways apparently does not necessarily generate traits that are generally more advantageous for the pathogen to grow and persist in human lungs. Mild courses of chronic infections with *P. aeruginosa* in CF come along with less lung damage than the severe courses. Late isolates from a mild course will grow in a CF lung that is close to the living conditions of an immunocompetent non-CF habitat with restricted nutrient conditions, whereas late isolates from a severe course colonize numerous niches of highly inflamed and remodeled tissue (Rosenow et al., 2015; Klockgether et al., 2018). *The owerwhelming amounts of nutrient- rich mucus present in the CF-lungs, further decreases the mobility of immune cells to different lung areas.* In response to bacterial invasion, the bodies own neutrophils release more proteases, which in turn leads to additional destruction of the host’s own cells and thus provides a source of amino acids and peptides (Quinn et al., 2019). In our experiments, isolates that are adapted to such conditions show a growth disadvantage when re-cultivated in the presence of PCLS from healthy lungs compared to the un-adapted early isolates.

Thus, the distinct fitness of intermediate and late *P. aeruginosa* CF isolates from mild and severe courses in our competition experiments could be attributed to the different ecological micro-niches in a close-to-normal and heavily destroyed CF lung, respectively (Camus et al., 2021). In other words, living conditions were substantially different for *P. aeruginosa* when growing in the lungs of mildly and severely affected CF hosts. This phenotypic difference could inherently not mimicked by our source of non-CF lung donors selected not to affect the outcome of the competition experiments. Please note that the investigated serial isolates represent the clinically most extreme courses of infection in our strain collection of 35 long-term courses (Cramer et al., 2012) leading to divergent modes of bacterial within-host evolution. For example, the acquisition of loss-of-function mutations was prominent during the fatal downhill course of infection, whereas a genetic gain of metabolic versatility was found to be typical for mild courses of infection. We chose hPCLS as an appropriate *ex vivo* model for our competition experiments because PCLS reflect the functional heterogeneity of lung tissue with all cell types present (Switalla et al., 2010) and thus has been widely used for drug testing (Liu et al., 2021), viral (Wronski et al., 2021) and bacterial infections (Dragan and Voth, 2021). The evaluation of the serial isolates in explanted CF lung tissue would be highly preferable, however, tissue from end-stage CF lung disease does not allow any *ex vivo* culture due to its strong microbial colonization (Carranza-Rosales et al., 2017). CF organoids (Kordes et al., 2019; Liu et al., 2020) or CF airway-on-chip (Ramalho et al., 2021; Nof et al., 2022) may be alternative options for forthcoming studies provided that the differentiation into a lung-like structure will become feasible.

In conclusion, the e*x vivo* competition experiments demonstrated that the patients’ disease status, particularly the stage of lung disease, was more relevant for the microevolution of *P. aeruginosa* than the bacterial clone type or genetic repertoire. The clinical relevance of the chronic airway infections with *P. aeruginosa* in CF is documented in patient registries by the time course of lung function, lung morphology scores and the outcome of culture-dependent diagnostics of respiratory secretions, respectively (Konstan et al., 2021; Muhlebach et al., 2021; Orenti et al., 2021). However, these biomarkers are not combined with the complex interface of host - pathogen interactions. On the other side, the microevolution of *P. aeruginosa* in CF lungs has already been thoroughly examined in its adaptive and evolutionary trajectories (Marvig et al., 2015; Blanchard and Waters, 2019; Camus et al., 2021), but we still miss the holistic view to intertwine bacterial omics data with features of the host. Our study may be taken as a memento to interpret the clinical significance of the airway infection with *P. aeruginosa* in the context of the individual habitat, inflammation and microbiome that generates a broad personalized range of microbial lifestyles, microbe – microbe and host-microbe interactions shaping the patient’s quality of life and prognosis (Bell et al., 2020).

## Data availability statement

The data presented in the study can be found in the **Supplementary Material** and online at the following link https://github.com/NinaCramer/results-competitive-fitness-mPCLS-hPCLS.git.

## Ethics statement

The studies involving human participants were reviewed and approved by Ethic committee Medical School Hannover Ethic approval number 2701-2015. The patients/participants provided their written informed consent to participate in this study. Ethical review and approval was not required for the animal study because since murine tissue was taken from dead animals only no ethic review/approval by local authorities was needed.

## Author contributions

The study was conceived and designed by NC and AM. Wet-lab experiments were mainly performed by NC, MLN, and LW. AM prepared the murine lungs. CS, OD, SW and AB provided protocols and the set-up for the adaptation of the PCLS to multi-marker competition experiments. DJ provided human lung tissue. MD prepared the amplicon sequencing libraries. Sequencing data were analyzed and compiled into data files, figures and tables by NC, MLN and SF. NC and BT wrote the manuscript and all authors commented and approved the submitted version.

## Funding

This work was funded by the Deutsche Forschungsgemeinschaft (DFG–SFB900/3–158989968 -A2 and Z1), the Bundesministerium für Bildung und Forschung (BMBF) - German Center for Lung Research (DZL) at the Hannover site (BREATH)- 82DZL002A1 and the German Center for Infection Research (DZIF) – TI 07.003.

## Acknowledgments

We cordially thank the DZL-supported Lung Pathology Group at Hannover Medical School for the provision of lung tissue and the team at Fraunhofer ITEM for generating PCLS from explanted human lung tissue. We thank Emma Spies for practical instructions to prepare PCLS during the initiation phase of the project. Sequencing at the Core Unit Genomics of Hannover Medical School is gratefully acknowledged.

## Conflict of interest

The authors declare that the research was conducted in the absence of any commercial or financial relationships that could be construed as a potential conflict of interest.

## Publisher’s note

All claims expressed in this article are solely those of the authors and do not necessarily represent those of their affiliated organizations, or those of the publisher, the editors and the reviewers. Any product that may be evaluated in this article, or claim that may be made by its manufacturer, is not guaranteed or endorsed by the publisher.

## Supplementary material

Supplementary Figure 1 Outcome of the second biological replicate of the competitive fitness experiments of 12 longitudinal courses in murine (left) and human PCLS (right). Relative growth changes of serial *P. aeruginosa* isolates during competitive fitness experiments are compared at 4 h and 8 h, respectively. Severe longitudinal courses are labeled with red, mild courses with blue letters. Each serial isolate is differentiated by color sorted by increasing colonization time of the clone in the patient’s lungs: *P. aeruginosa* strains marked without a black outer ring were recovered at such low quantities that the strain-specific SNP counts were classified as ‘detectable, but not quantifiable’ (see Material and methods section). The amplicon sequence data of the isolates of course B did not pass the quality control and hence the plots were intentionally omitted. The axis labeling as well as the scaling and the color of the isolates are explained in more detail in **Supplementary Dataset S1** showing all plots at higher resolution.

Supplementary Figure 2 Relative proportions of the biological replicate of serial isolates during competitive growth in murine (left) and human PCLS (right) after 4 hours and 8 hours. For each serial isolate of the twelve longitudinal courses, its fraction in the clonal community at the time points 0 h, 4 h and 8 h is shown. Longitudinal courses are differentiated by a bold letter (severe courses in red; mild courses in blue). Each serial isolate is depicted by color sorted by increasing colonization time of the clone in the patient’s lungs. For more detailed information see **Supplementary Dataset S1**.

Supplementary Data Sheet 1 Step-by-step explanation of the evaluation of the competition experiments in murine and human PCLS.

Supplementary Table 1 Data about *P. aeruginosa* isolates (clone, isolate, CF patient’s age, airway colonization time) and biomaterial (patients’ age, gender, diagnosis) chosen for hPCLS.

Supplementary Table 2 Tests for normal distribution and variance homogeneity. To verify if the values are normally distributed, ANOVA as well as Kolmogorov-Smirnov were used. Variance homogeneity was checked *via* Levene test.

Supplementary Table 3 Comparability of technical and biological replicates.

Supplementary Table 4 Statistical analysis. Statistical tests for mPCLS and hPCLS are separated by different data sheets. Groups of the first singletons, duos, trios, quartets, quintets and sextets were each compared to the sets of remaining isolates. All isolates were distributed into three different groups (early, intermediate and late isolates) depending on colonization time (see **Table S5**) and tested against each other. In case of statistics with more than two groups Kruskal-Wallis was used, all two-group comparisons were executed with Wilcoxon signed rank tests. All p-values are Bonferroni corrected and marked in red if mentioned in the body of the manuscript.

Supplementary Table 5 Distributions of clonal *P. aeruginosa* isolates separated by colonization time into three groups. **(A)** shows the results for mPCLS, **(B)** the results for hPCLS experiments. All sequential isolates were separated into three different groups depending on the colonization time. First isolates belonging to the first quarter of colonization time, the late isolates to the last quarter. All isolates within the inner quartiles were classified as intermediate isolates. For statistical analysis Kruskal-Wallis was used.
