# Supplementary figures and images for: Competitive fitness of Pseudomonas aeruginosa isolates in human and murine precision-cut lung slices
Source: Front Cell Infect Microbiol. 2022 Aug 23;12:992214. doi: 10.3389/fcimb.2022.992214 (PMC9446154; doi:10.3389/fcimb.2022.992214)

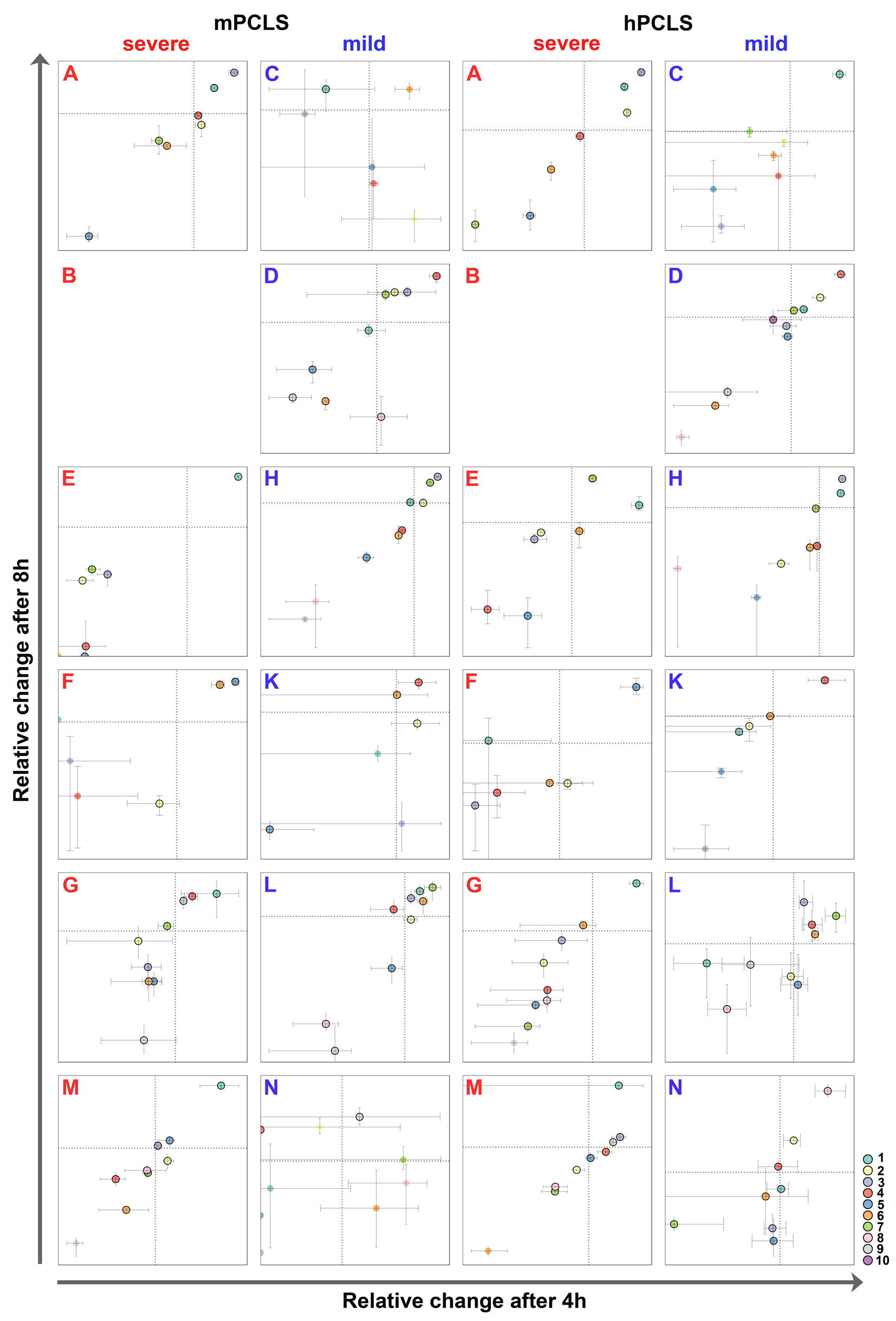

Supplement: Supplementary Figure 1 — Outcome of the second biological replicate of the competitive fitness experiments of 12 longitudinal courses in murine (left) and human PCLS (right). Relative growth changes of serial P. aeruginosa isolates during competitive fitness experiments are compared at 4 h and 8 h, respectively. Severe longitudinal courses are labeled with red, mild courses with blue letters. Each serial isolate is differentiated by color sorted by increasing colonization time of the clone in the patient’s lungs: P. aeruginosa strains marked without a black outer ring were recovered at such low quantities that the strain-specific SNP counts were classified as ‘detectable, but not quantifiable’ (see Material and methods section). The amplicon sequence data of the isolates of course B did not pass the quality control and hence the plots were intentionally omitted. The axis labeling as well as the scaling and the color of the isolates are explained in more detail in Supplementary Dataset S1 showing all plots at higher resolution. [file Image_1.tif]

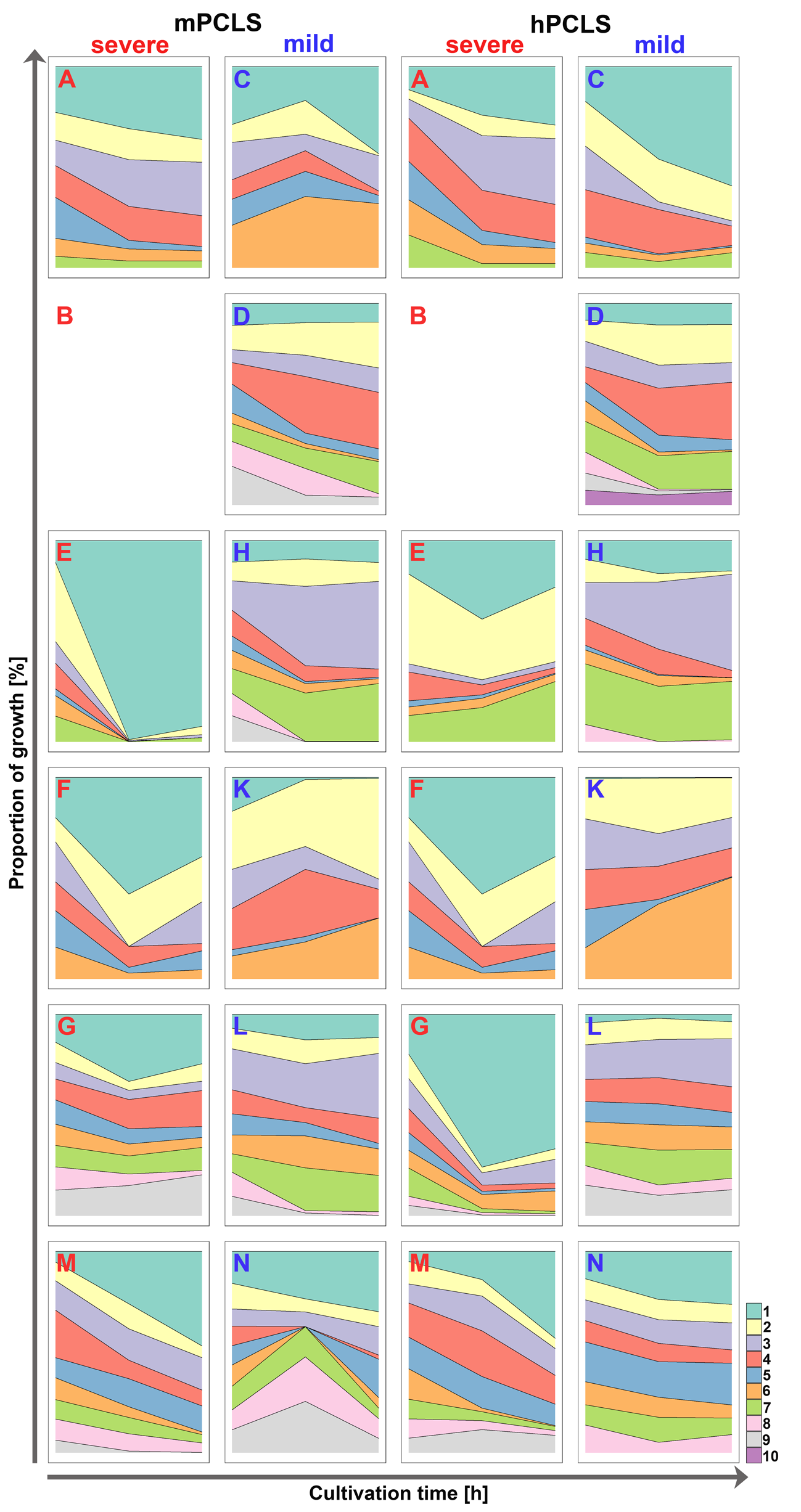

Supplement: Supplementary Figure 2 — Relative proportions of the biological replicate of serial isolates during competitive growth in murine (left) and human PCLS (right) after 4 hours and 8 hours. For each serial isolate of the twelve longitudinal courses, its fraction in the clonal community at the time points 0 h, 4 h and 8 h is shown. Longitudinal courses are differentiated by a bold letter (severe courses in red; mild courses in blue). Each serial isolate is depicted by color sorted by increasing colonization time of the clone in the patient’s lungs. For more detailed information see Supplementary Dataset S1 . [file Image_2.tif]
