# Supplementary material for: Competitive fitness of Pseudomonas aeruginosa isolates in human and murine precision-cut lung slices
Source: Front Cell Infect Microbiol. 2022 Aug 23;12:992214. doi: 10.3389/fcimb.2022.992214 (PMC9446154; doi:10.3389/fcimb.2022.992214)

Supplementary dataset E2

Read me:

This dataset is intended to explain the two different forms of presentation of the competition results to the reader. In order to present all results in a complex manner on one page, a highly condensed version was chosen in the manuscript with the help of Figures 2 and 3. On the following slides, all courses are shown again in both display forms at a larger resolution and with axes and legends. On the first slide, the first form of representation (Figure 2) is shown and explained using the example of course A. It shows the relative changes after 4h and after 8h in relation to the 0h point in time. Slide 2 shows the representation of the relative growth proportions of each serial isolate over the competition period, exemplarily for course A. Both forms of representation are then shown individually for all courses on the following slides. Both biological replicates and the results in mPCLS and hPCLS are shown next to each other on the same slide.

Example for the graphical representation of the outcome of the competitive fitness experiments

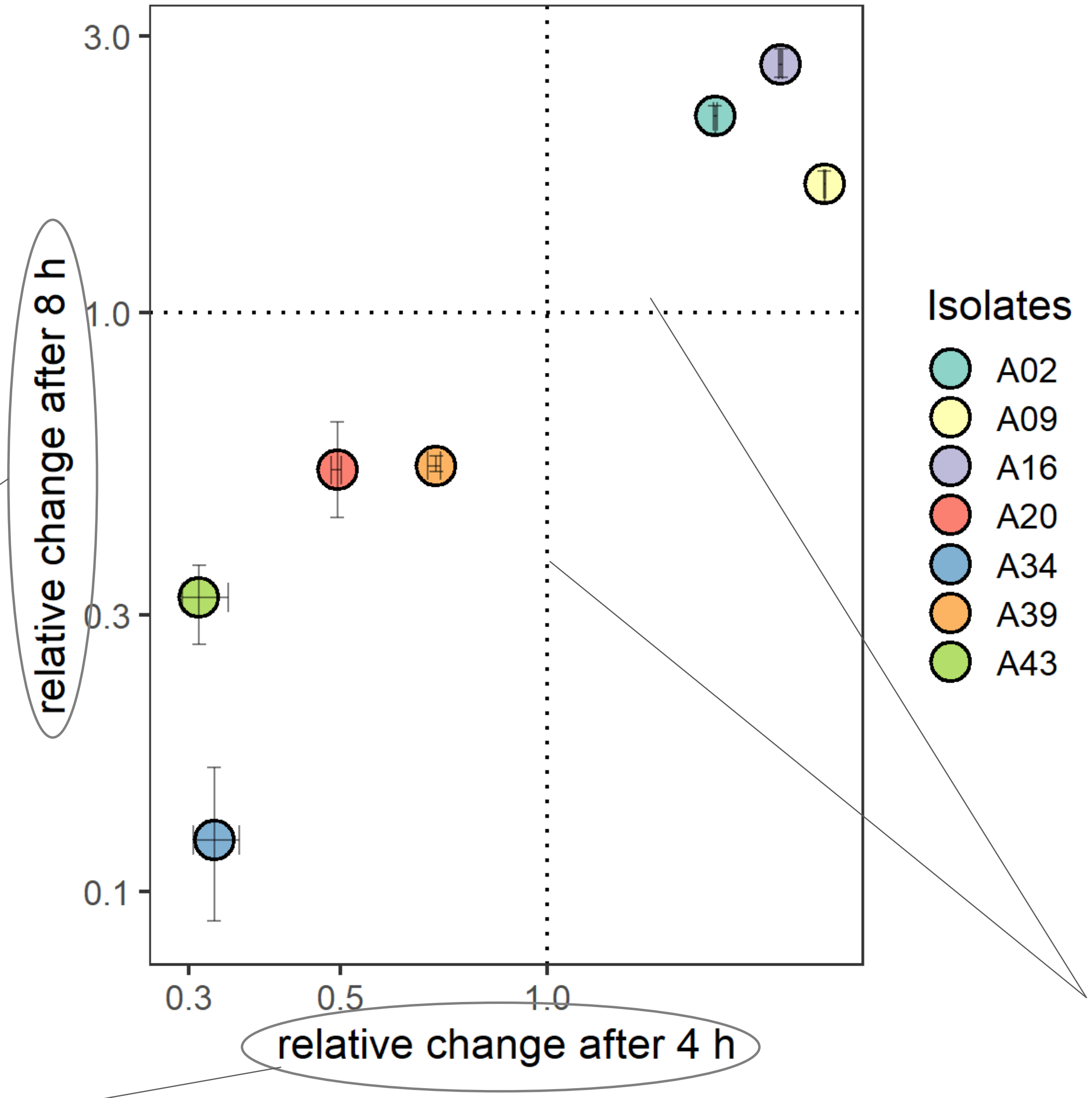

$$\frac{growth\ after\ 8h\ [\%]}{growth\ at\ the\ beginning\ [\%]}$$

$$\frac{growth\ after\ 4h\ [\%]}{growth\ at\ the\ beginning\ [\%]}$$

Serial isolates differ by a specific colour depending on the colonisation time

The line at 1 marks the point at which the growth of an isolate has stagnated compared to 0h (has remained at the same level).

→ Winner strains are in the upper right quarter of the plot

Example for the graphical representation of the relative proportions of serial isolates during competitive growth

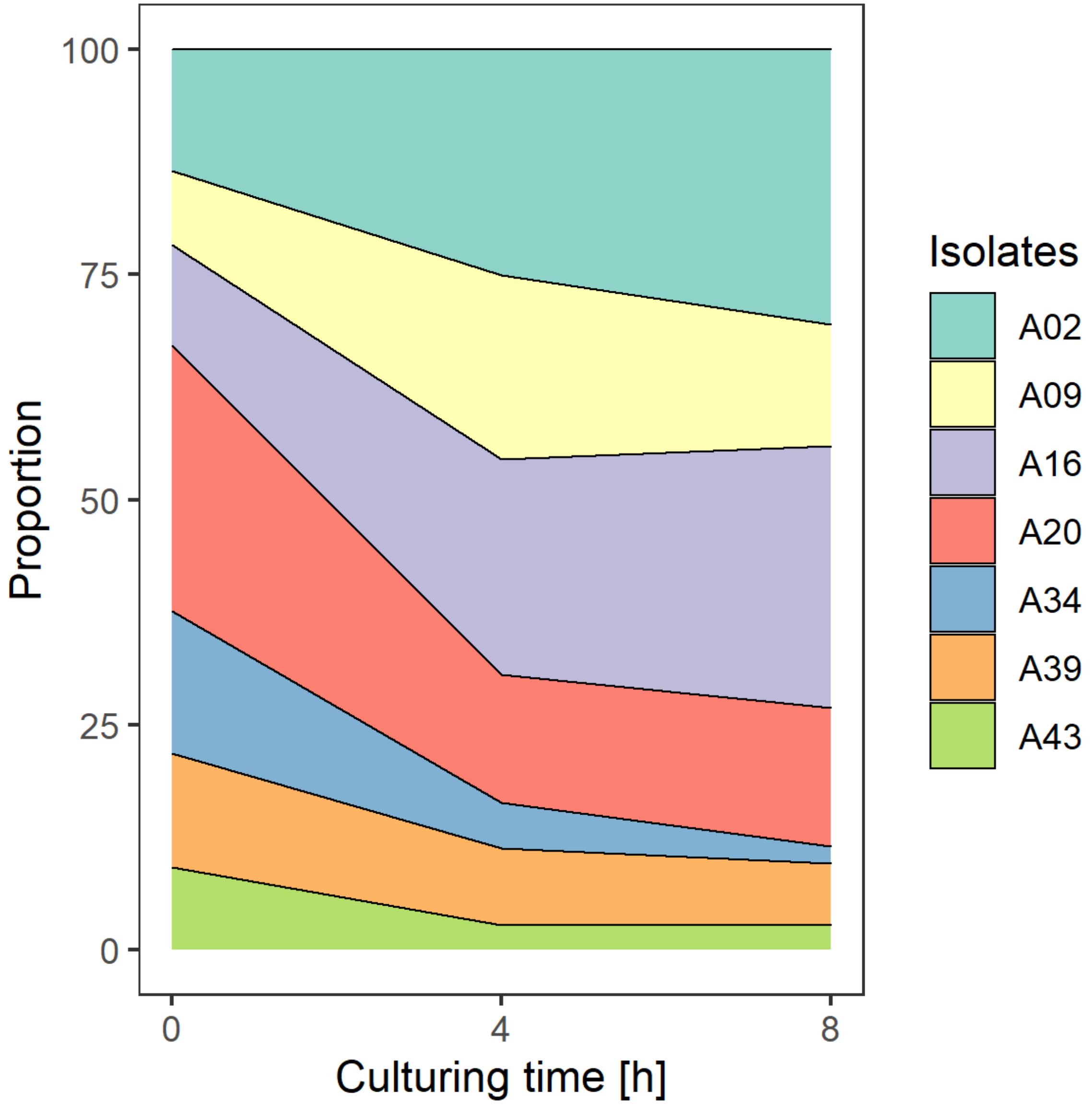

Proportion of each serial isolate; sum of isolates were normalized to 100%

Serial isolates differ by a specific colour depending on the colonisation time

Samples were taken after 4h and 8h of competitive growth

Course A

human | murine

1st replicate

2nd replicate

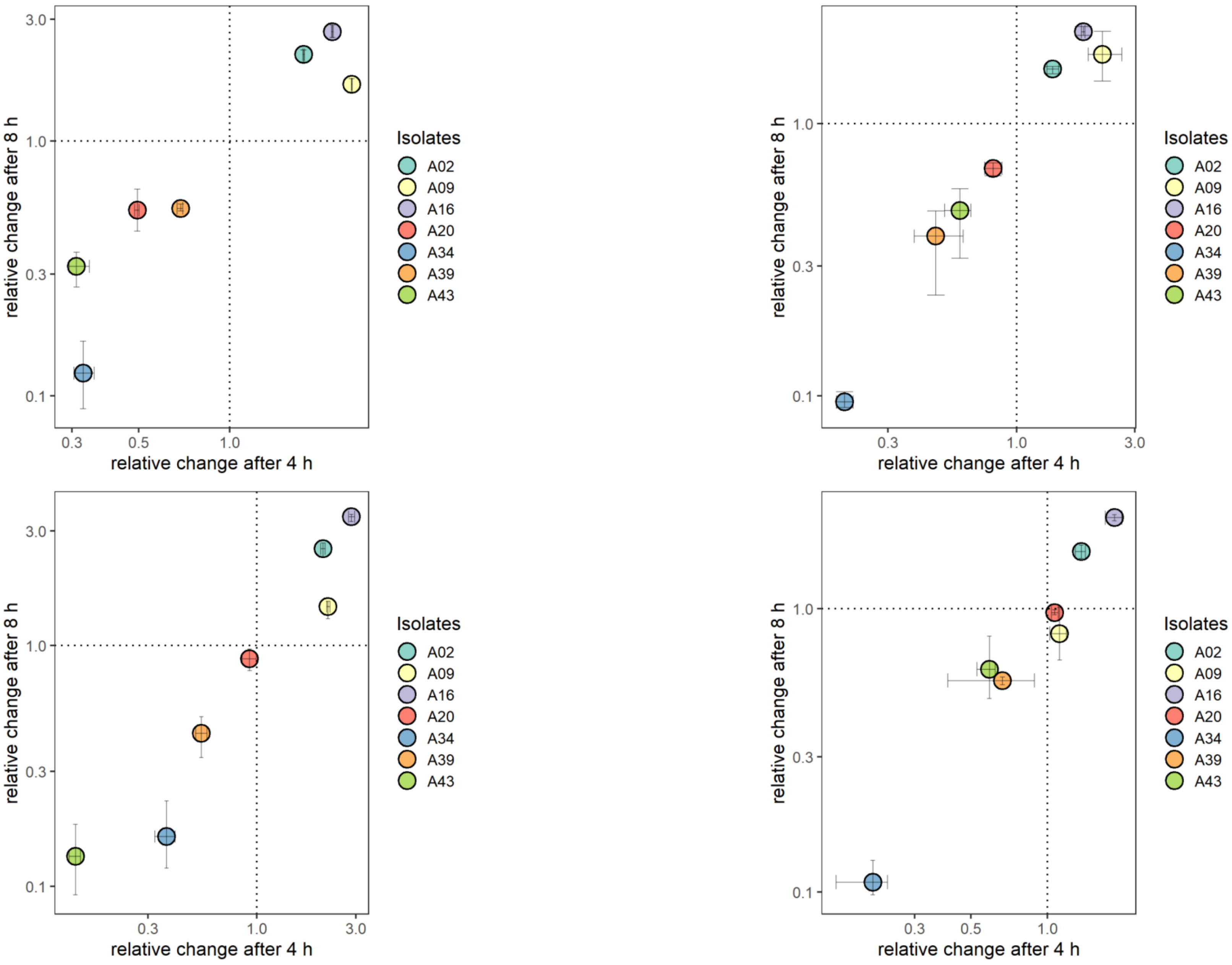

Course B

human

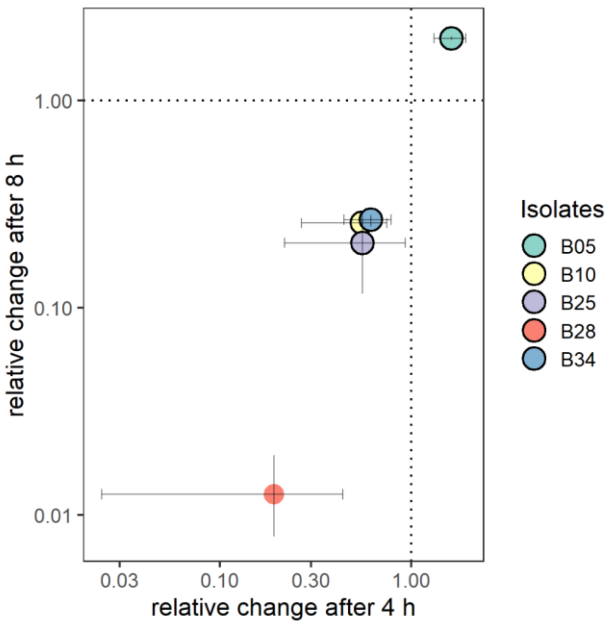

murine

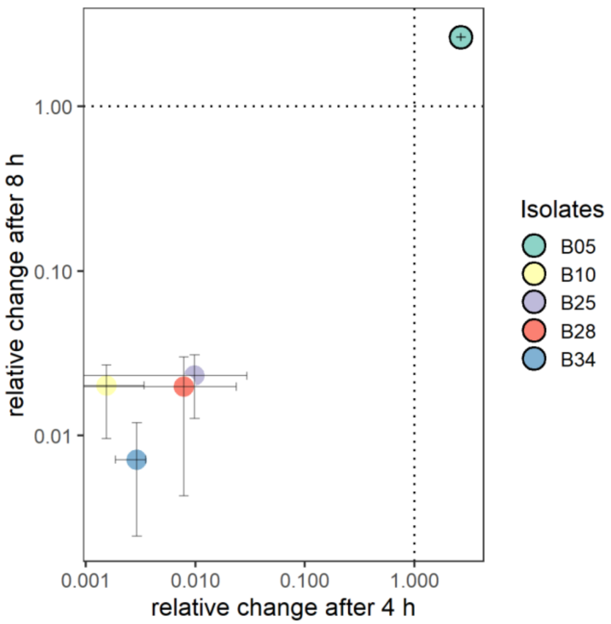

1st replicate

2nd replicate

The second biological replicate of course B was removed from the further statistical calculation / evaluation as well as for the graphic representation of the results, since only 4 isolates corresponded past the quality control.

Course C

human | murine

1st replicate

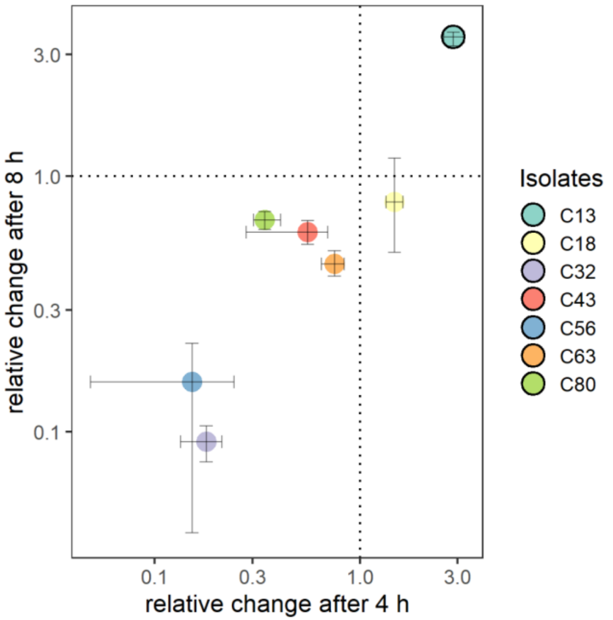

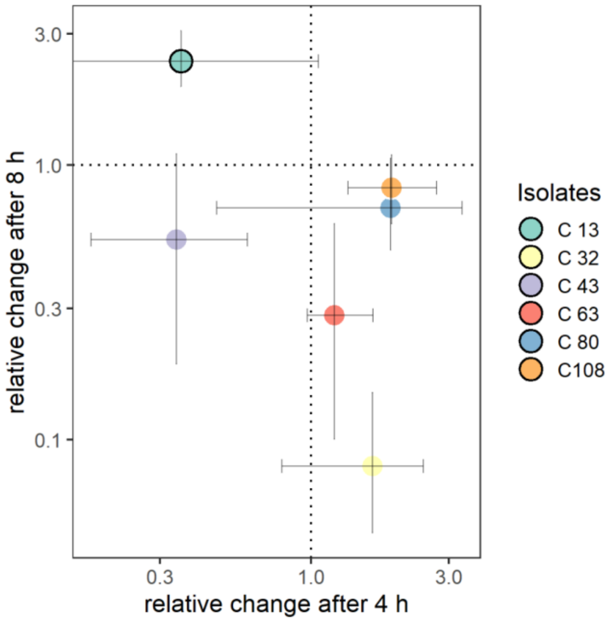

2nd replicate

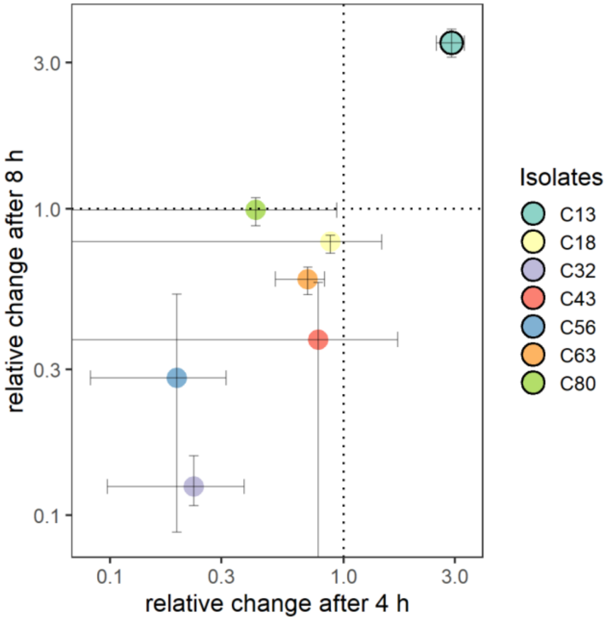

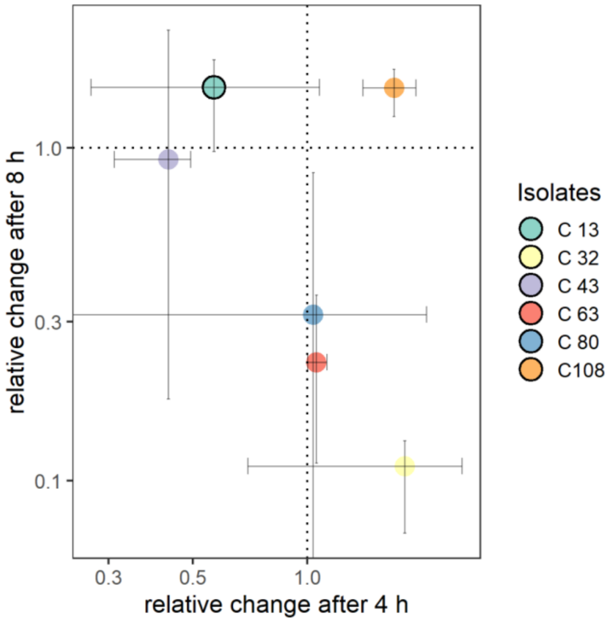

Course D

human

murine

1st replicate

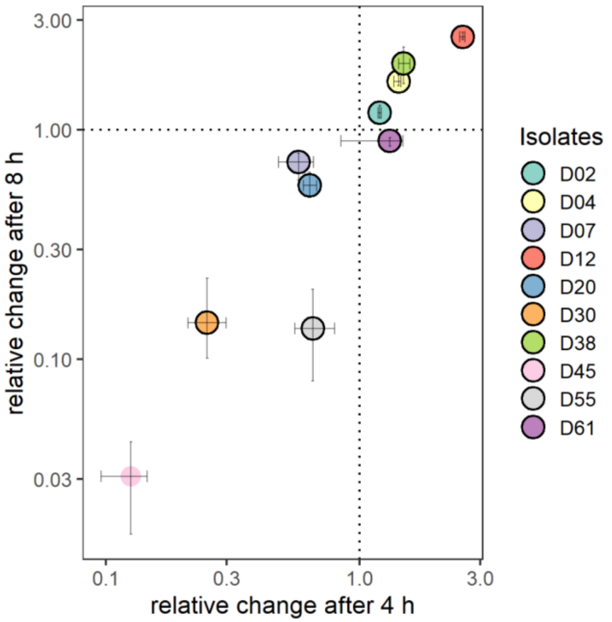

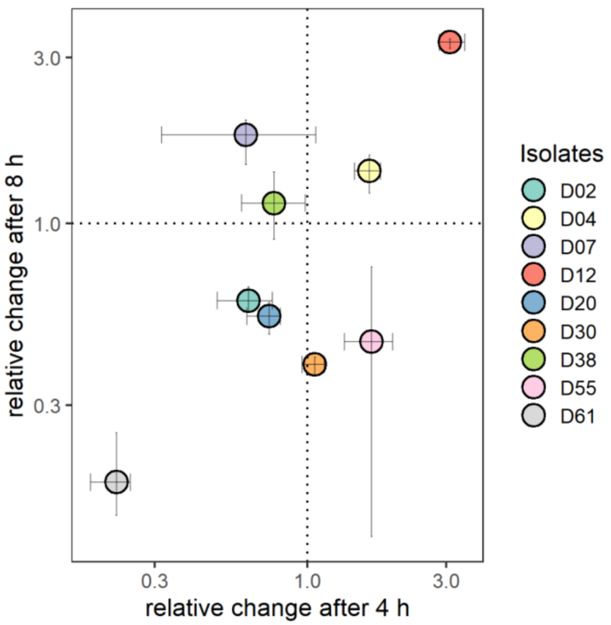

2nd replicate

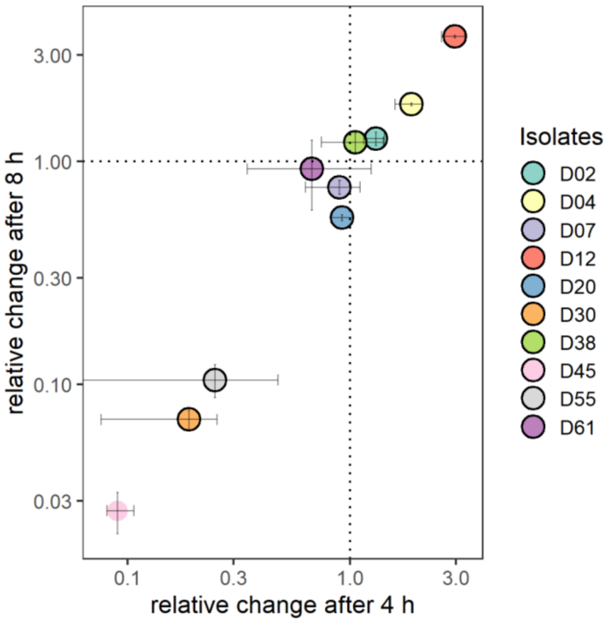

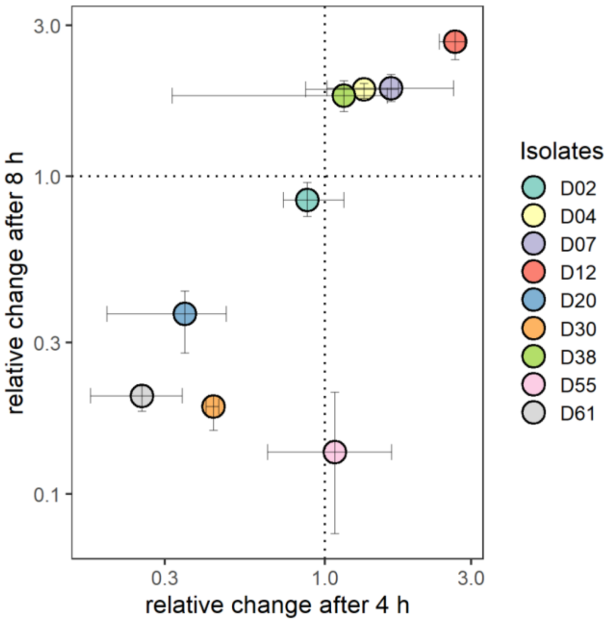

Course E

human | murine

1st replicate

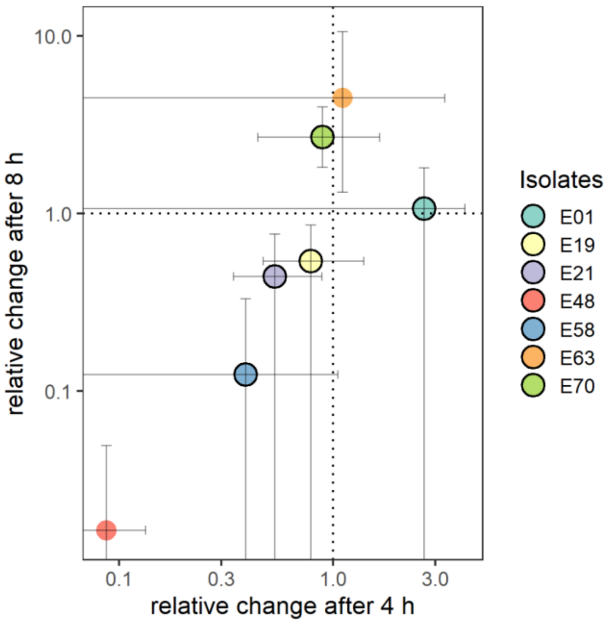

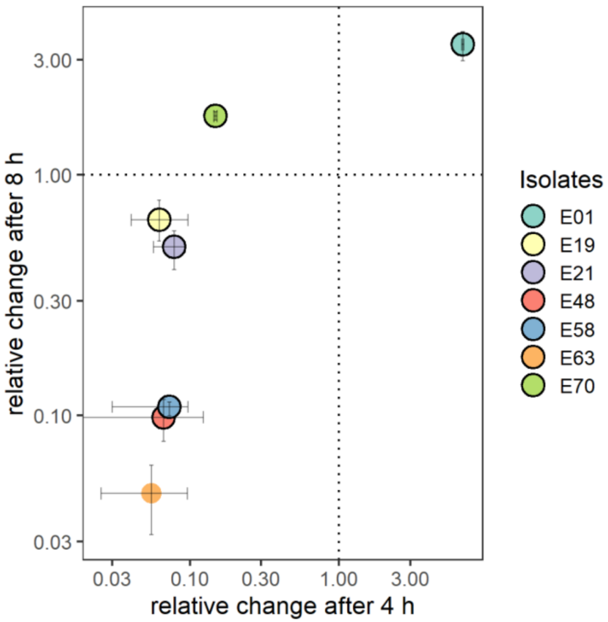

2nd replicate

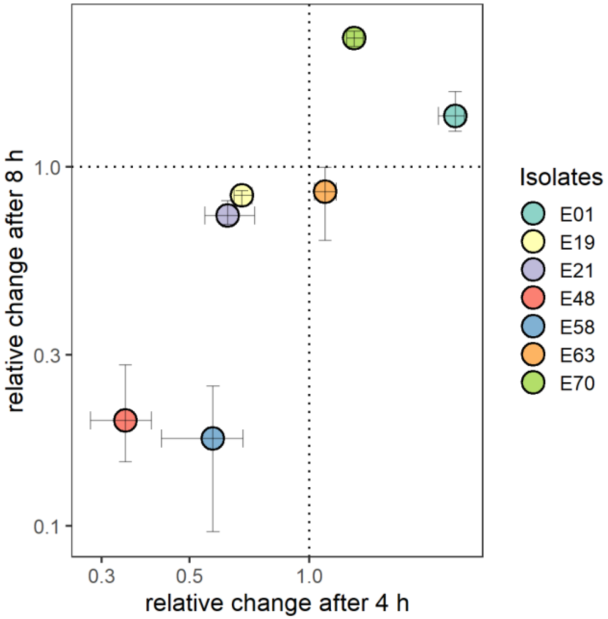

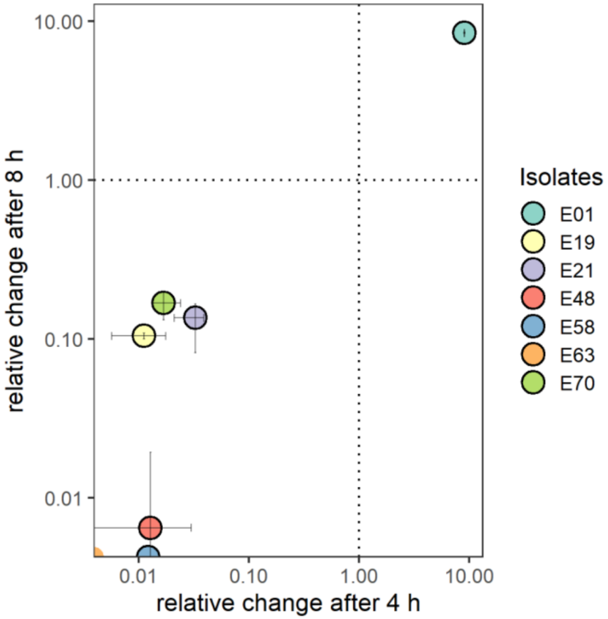

Course F

human | murine

1st replicate

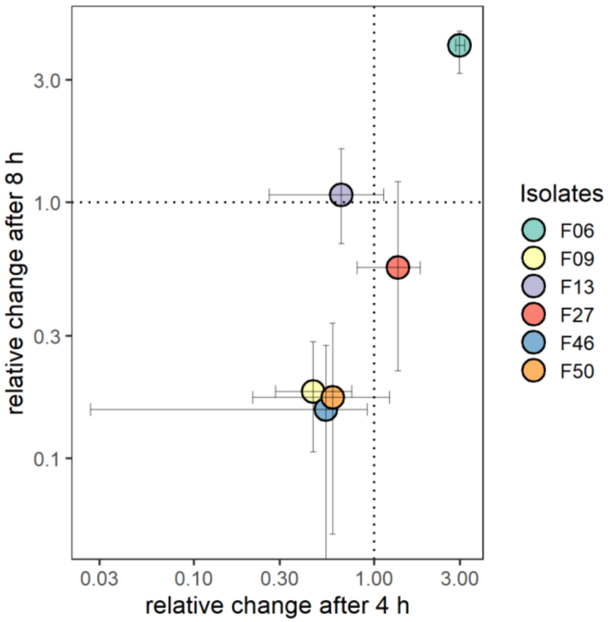

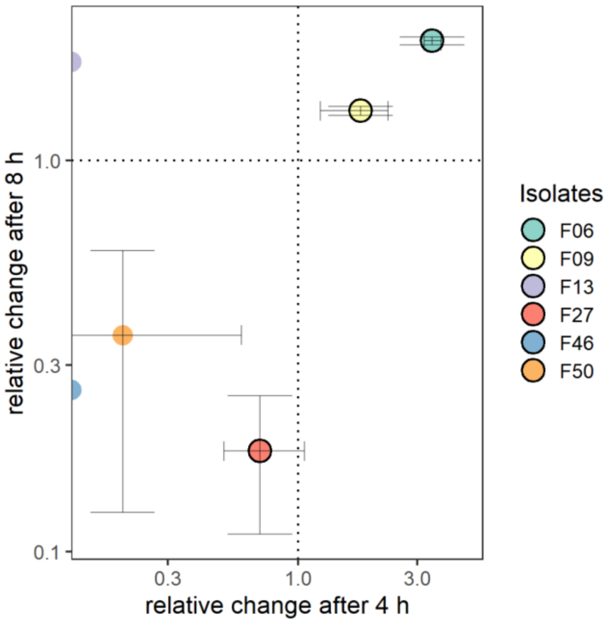

2nd replicate

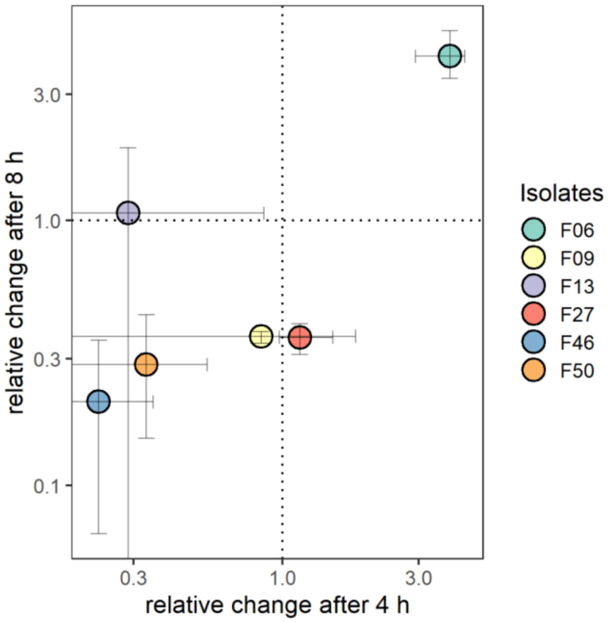

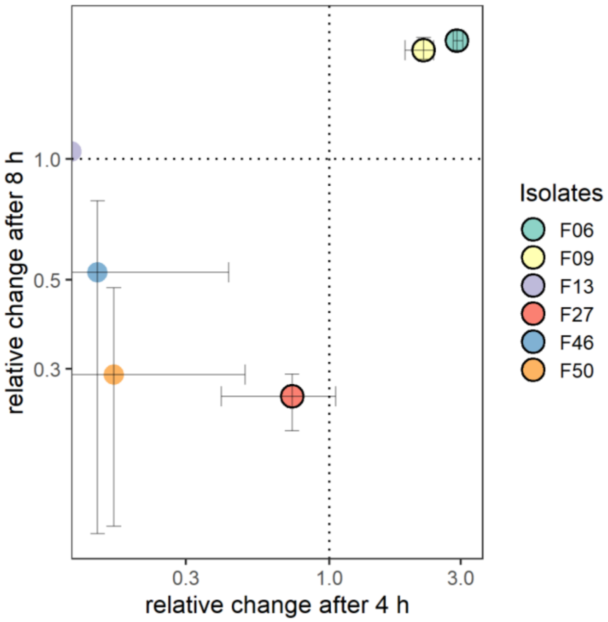

Course G

human

murine

1st replicate

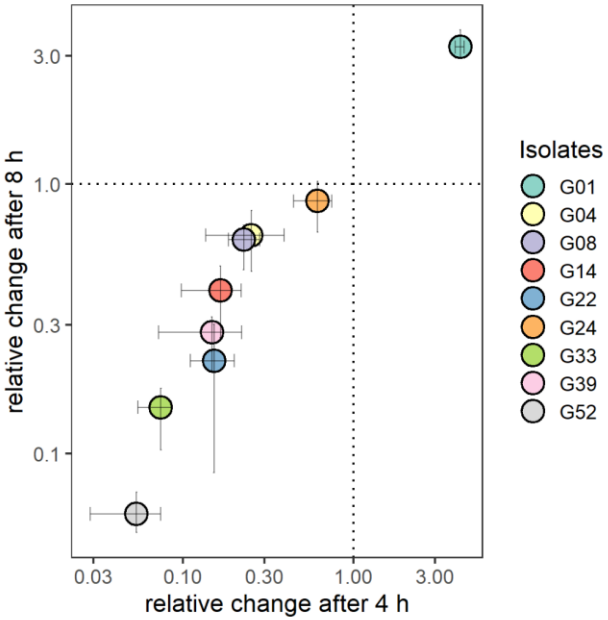

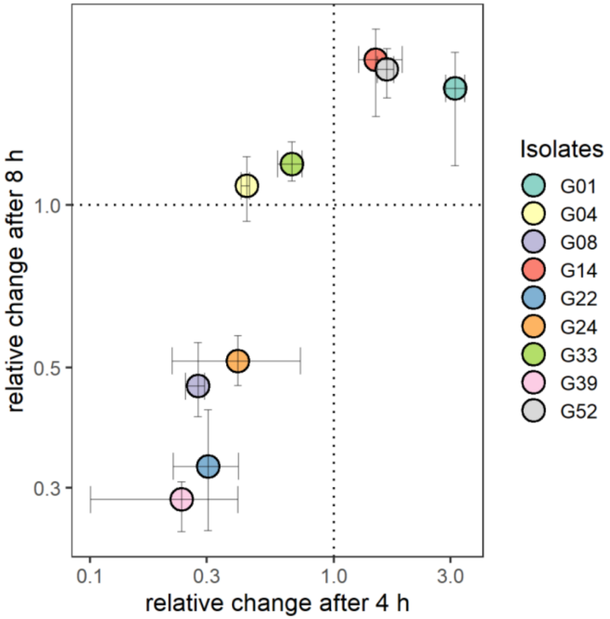

2nd replicate

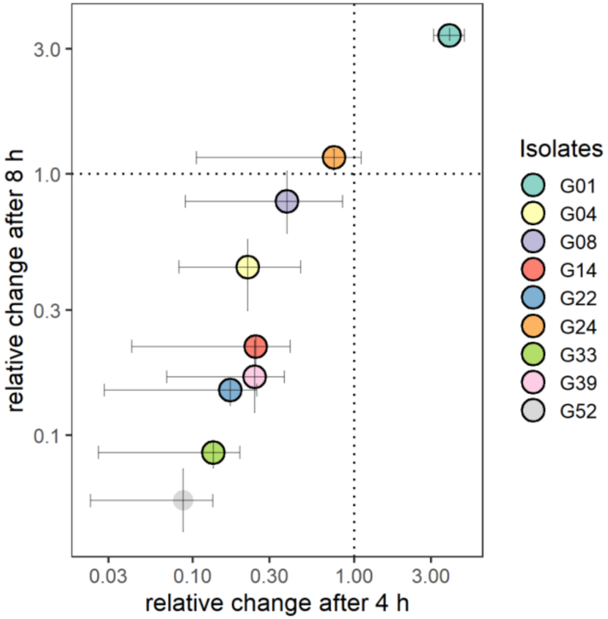

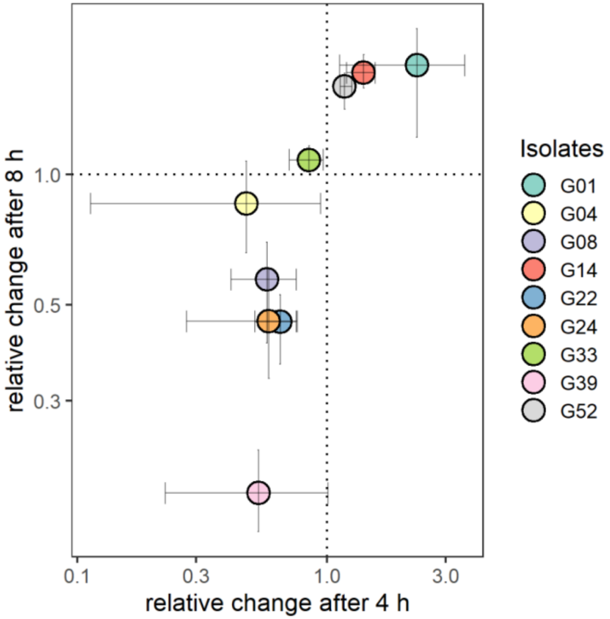

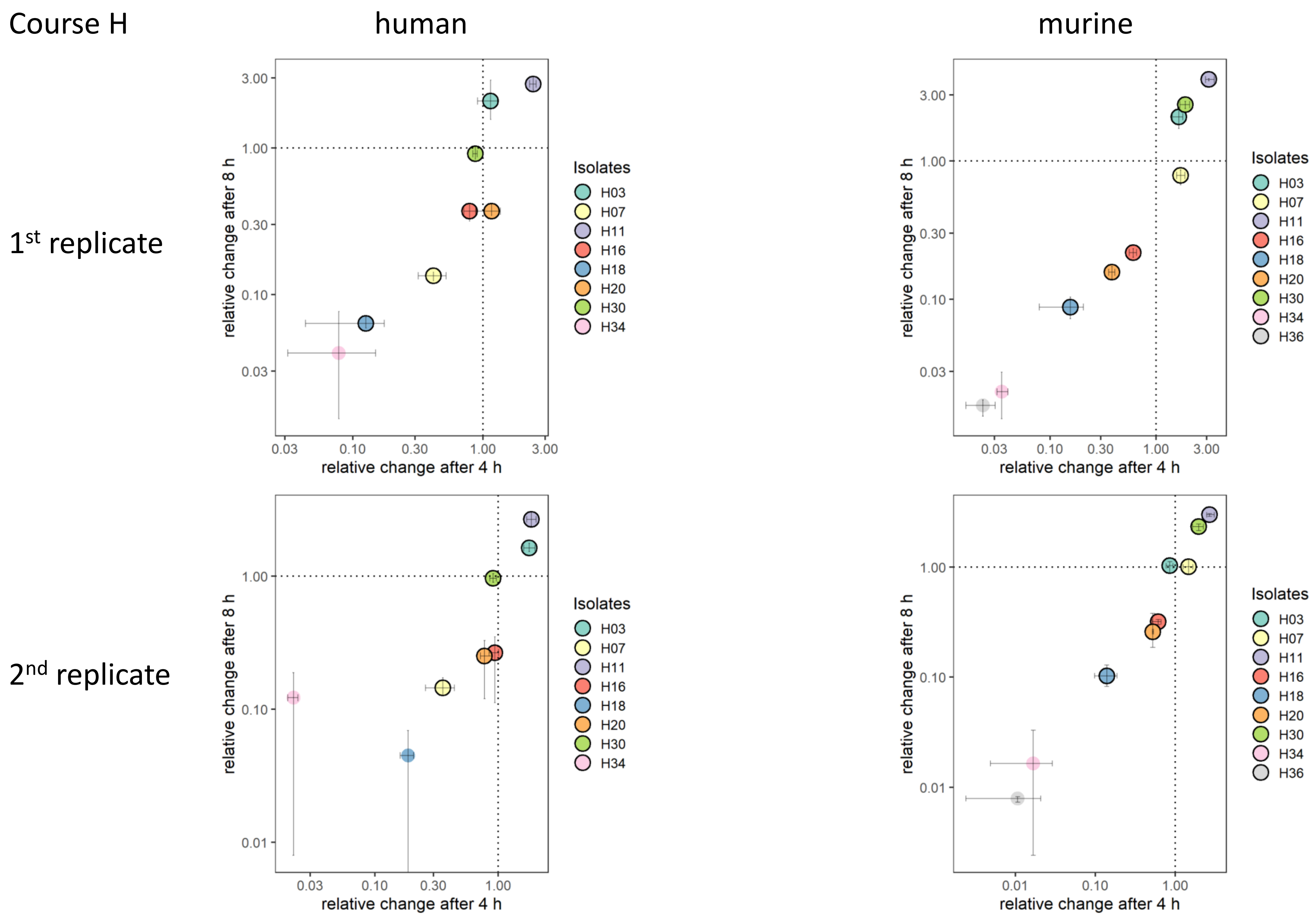
Course H
human
murine
1st replicate
2nd replicate
relative change after 8 h
relative change after 4 h
3.00
1.00
0.30
0.10
0.03
0.01
Isolates
H03
H07
H11
H16
H18
H20
H30
H34
H36

Course K

human

murine

1st replicate

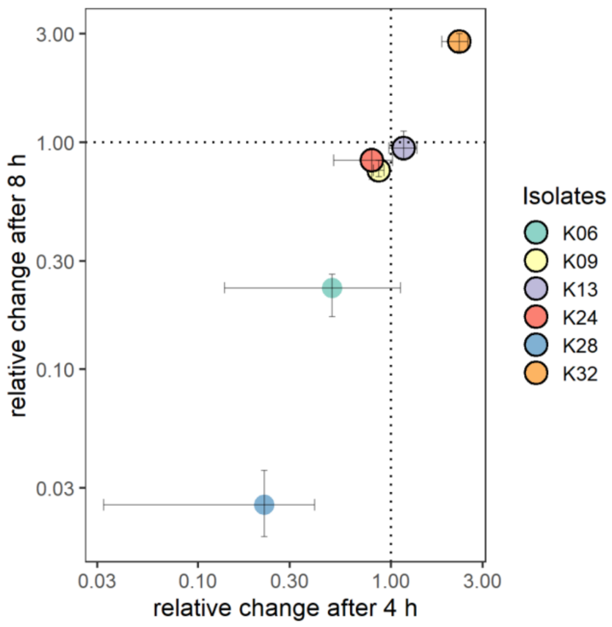

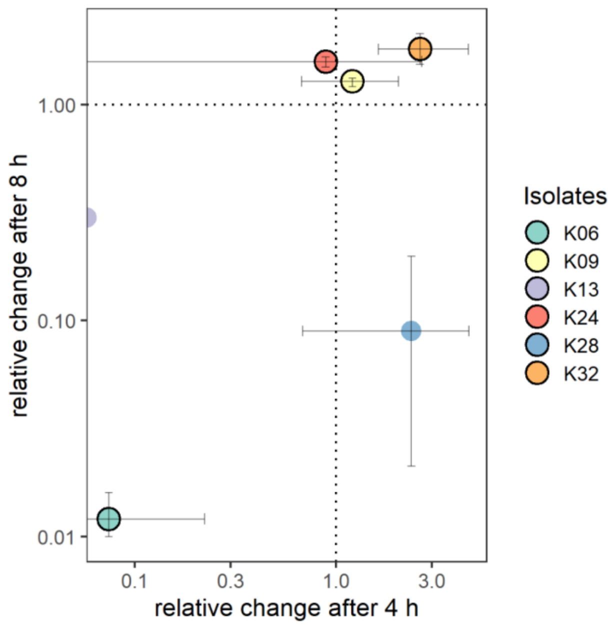

2nd replicate

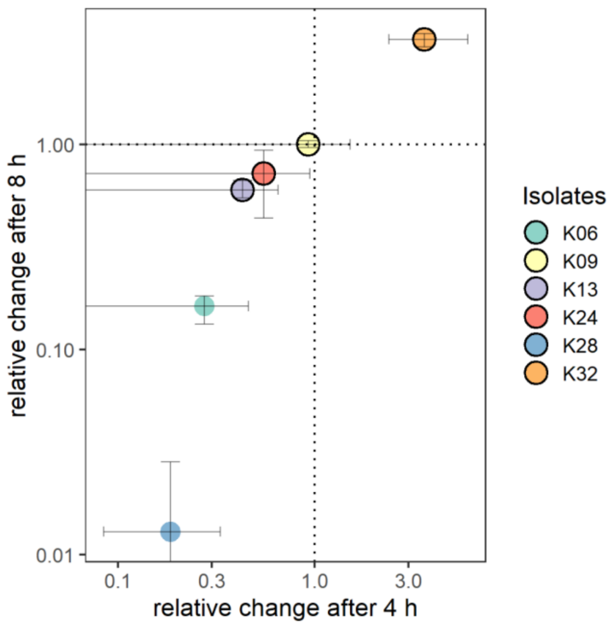

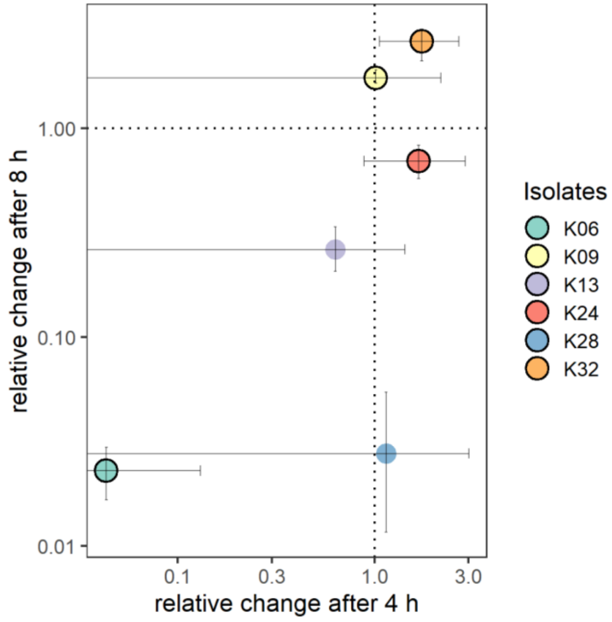

Course L

human | murine

1st replicate

2nd replicate

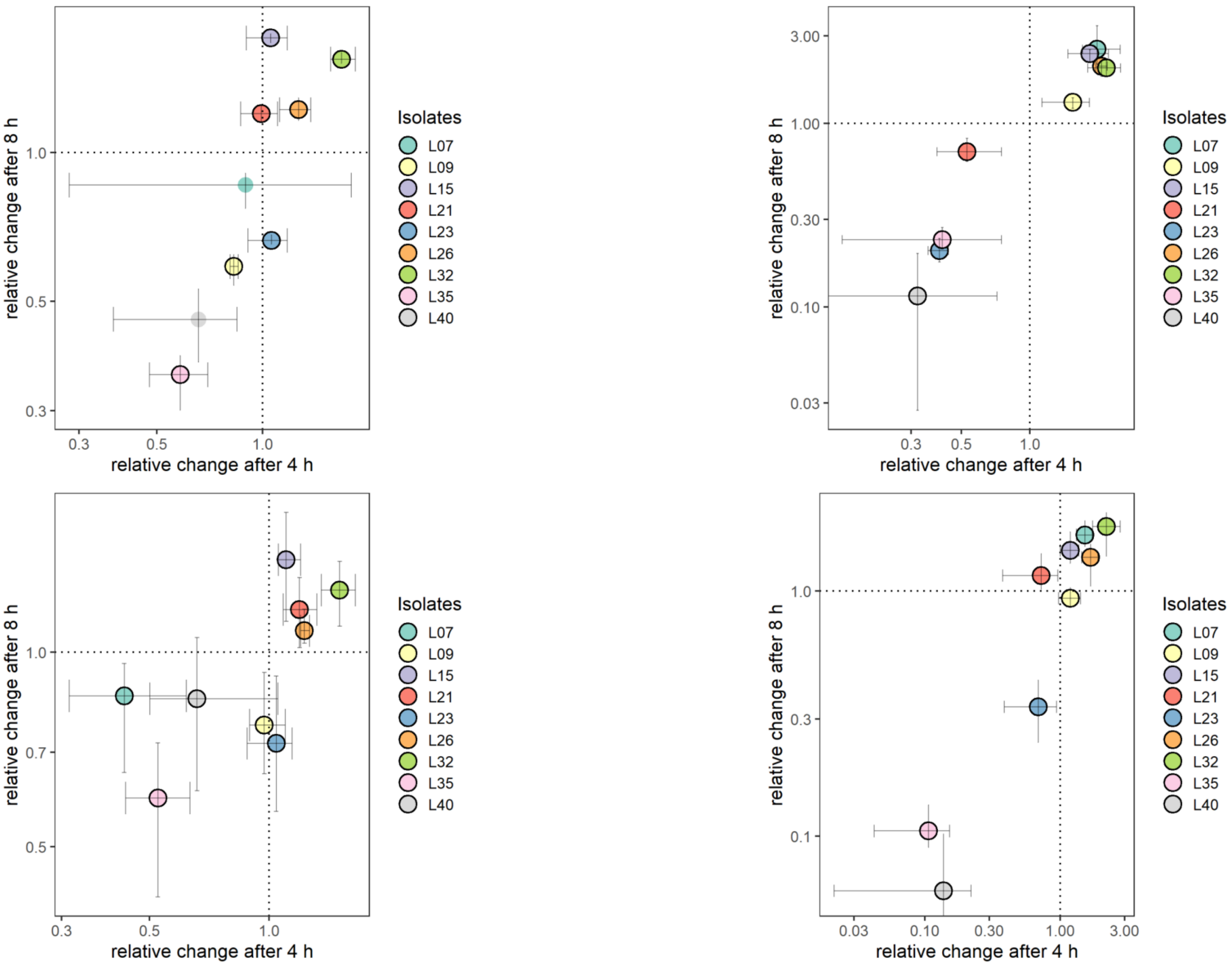

Course M

human

murine

1st replicate

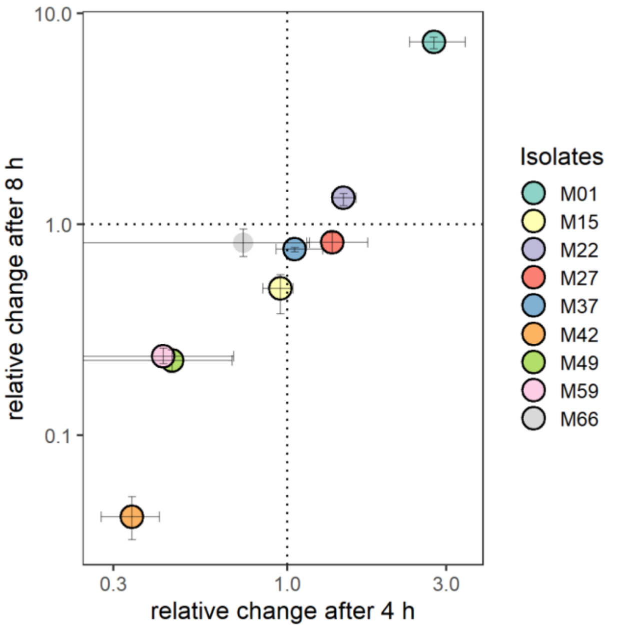

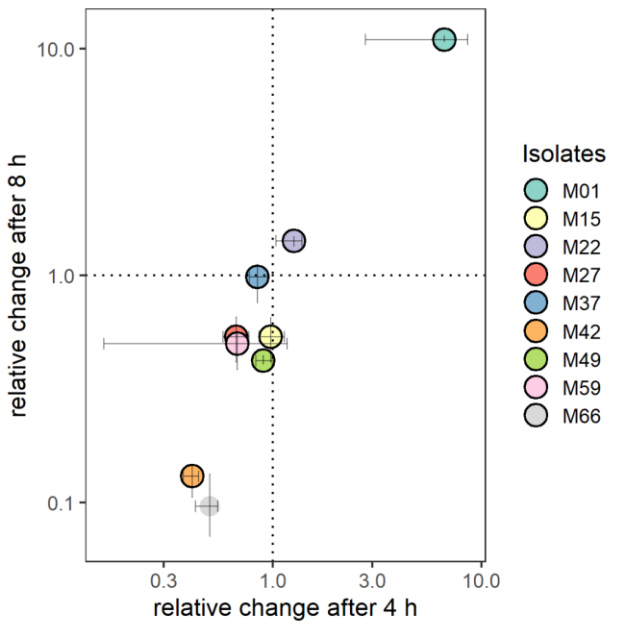

2nd replicate

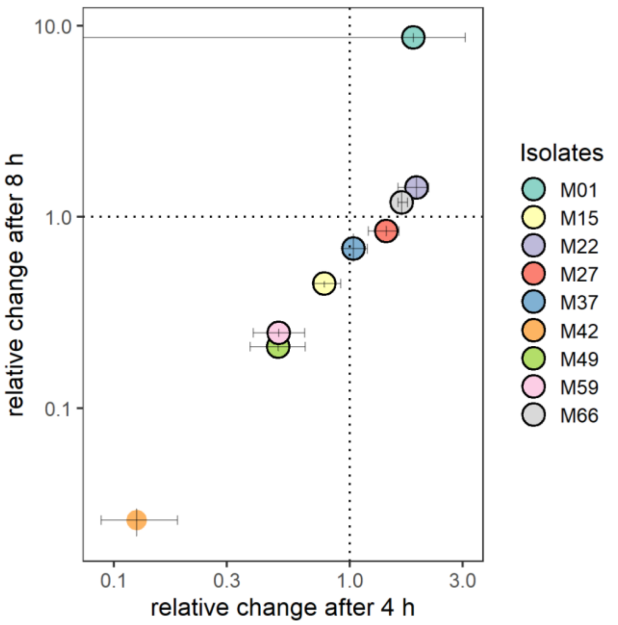

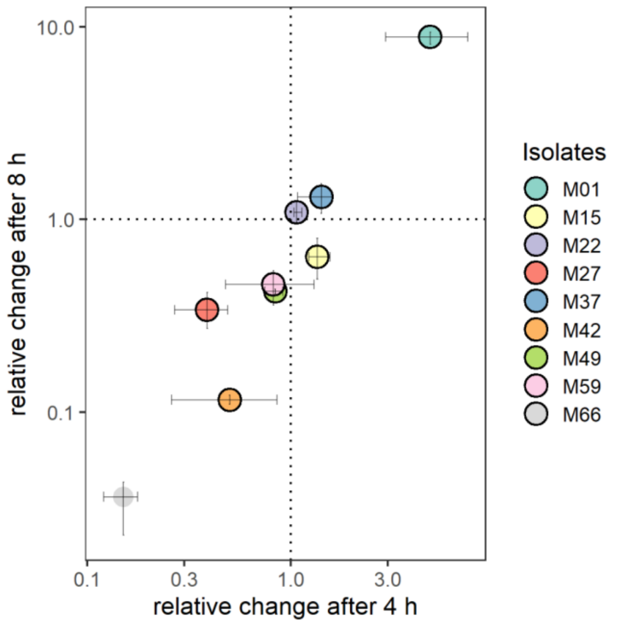

Course N

human | murine

1st replicate

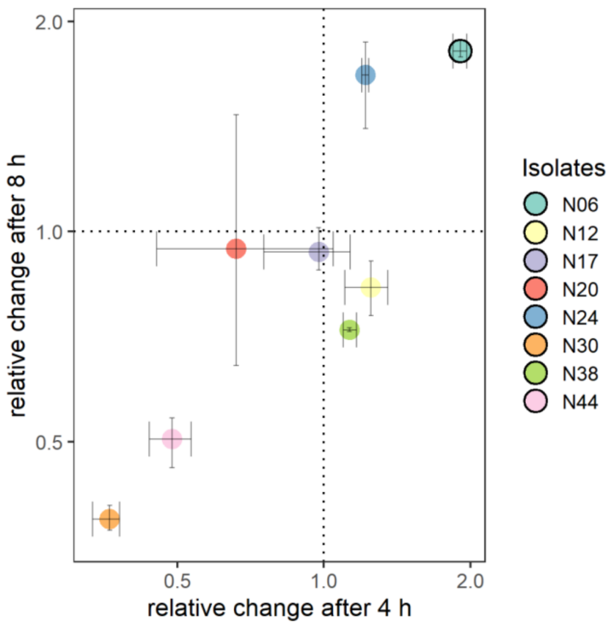

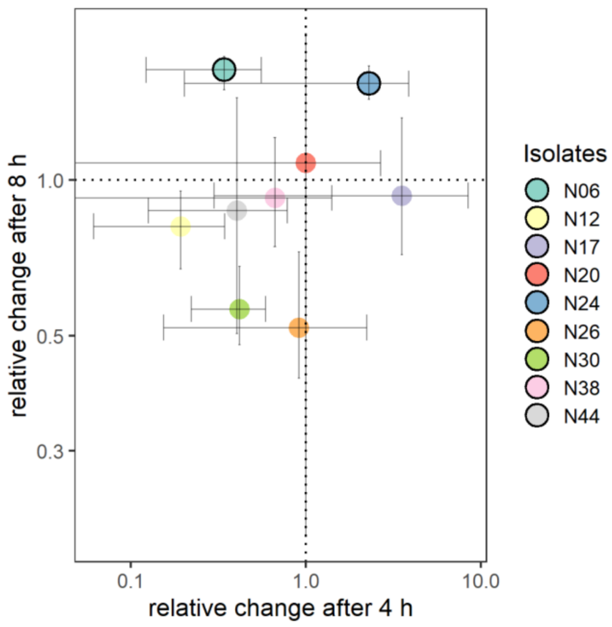

2nd replicate

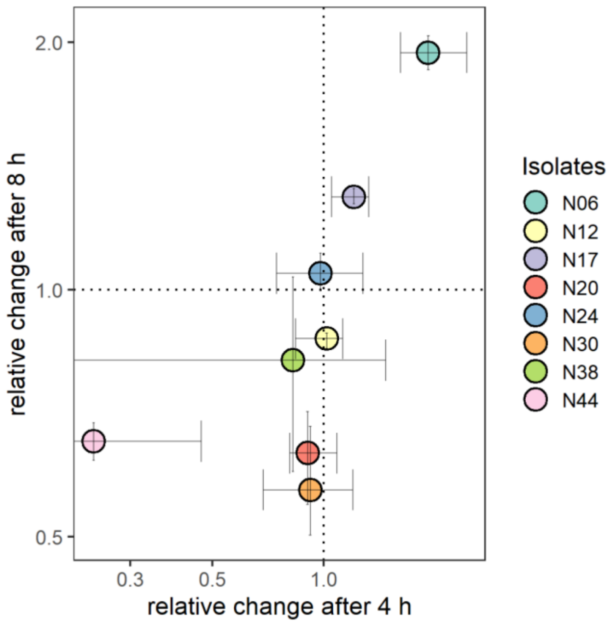

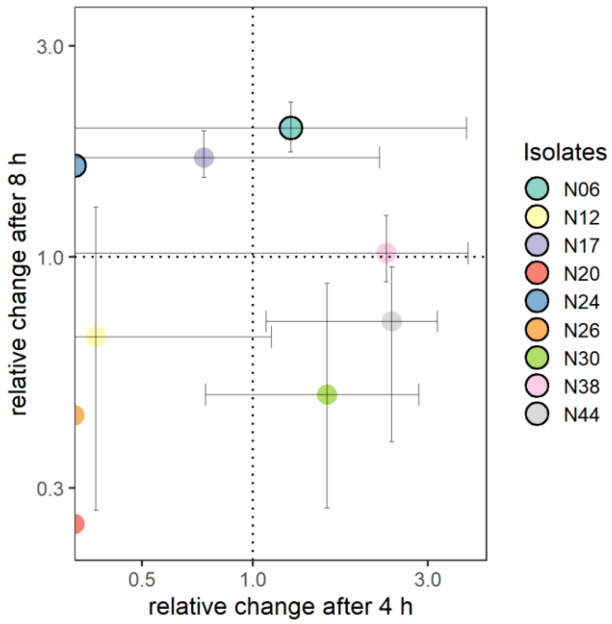

Course A

human | murine

1st replicate

2nd replicate

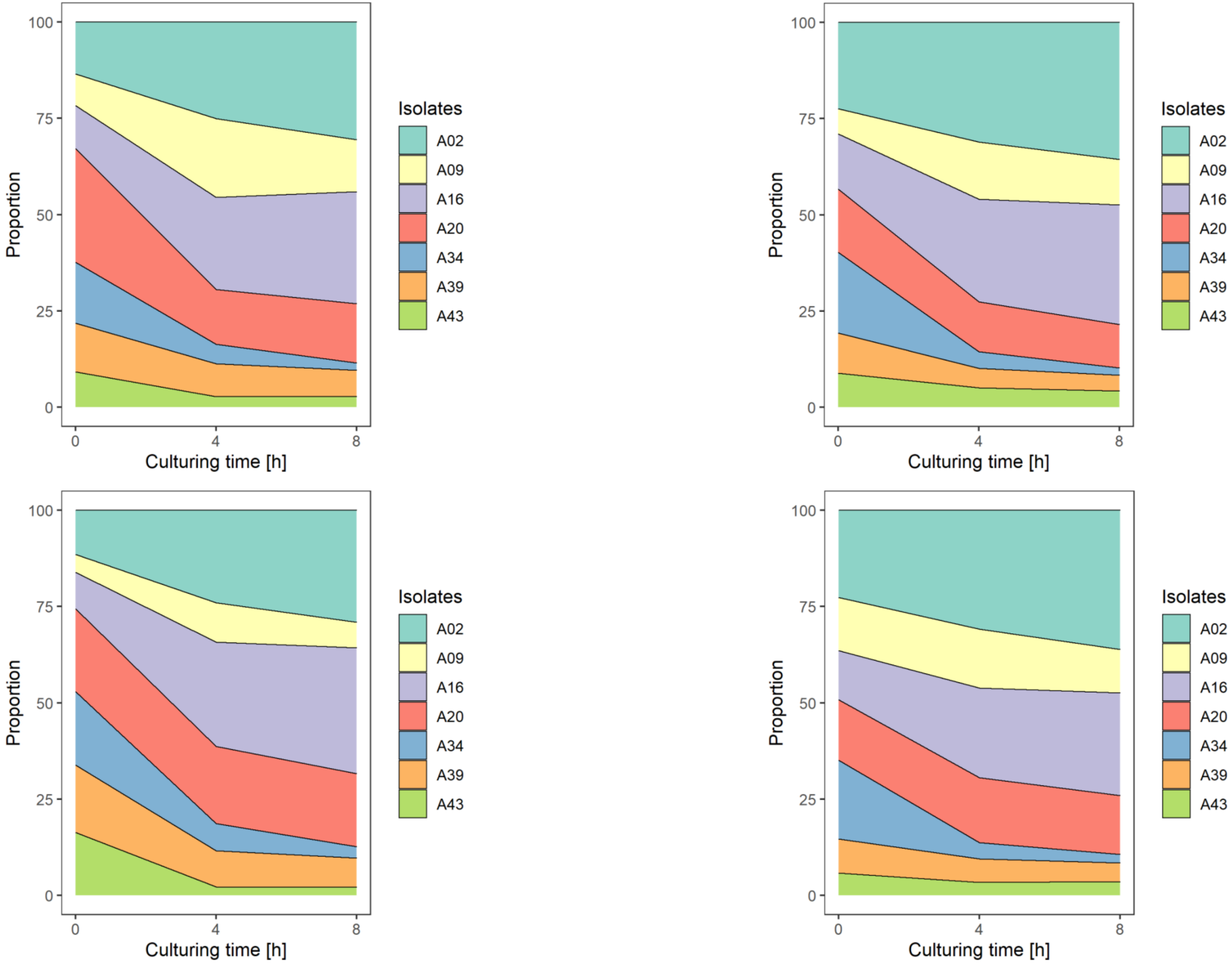

Course B

human

murine

1st replicate

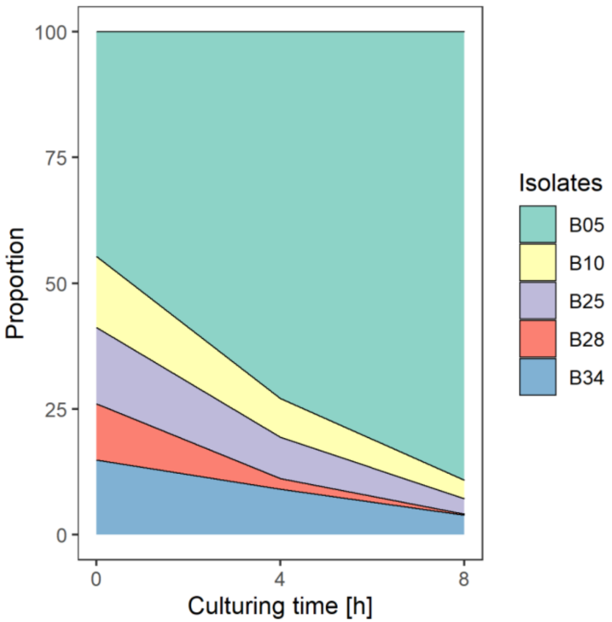

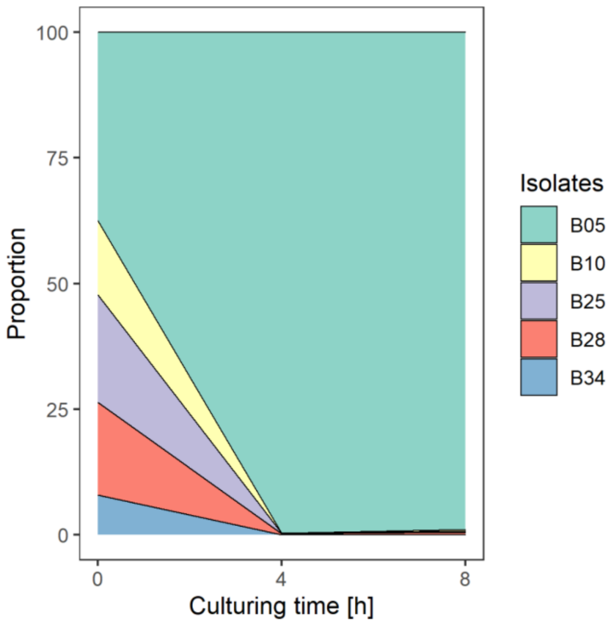

2nd replicate

The second biological replicate of course B was removed from the further statistical calculation / evaluation as well as for the graphic representation of the results, since only 4 isolates corresponded past the quality control.

Course C

human

murine

1st replicate

2nd replicate

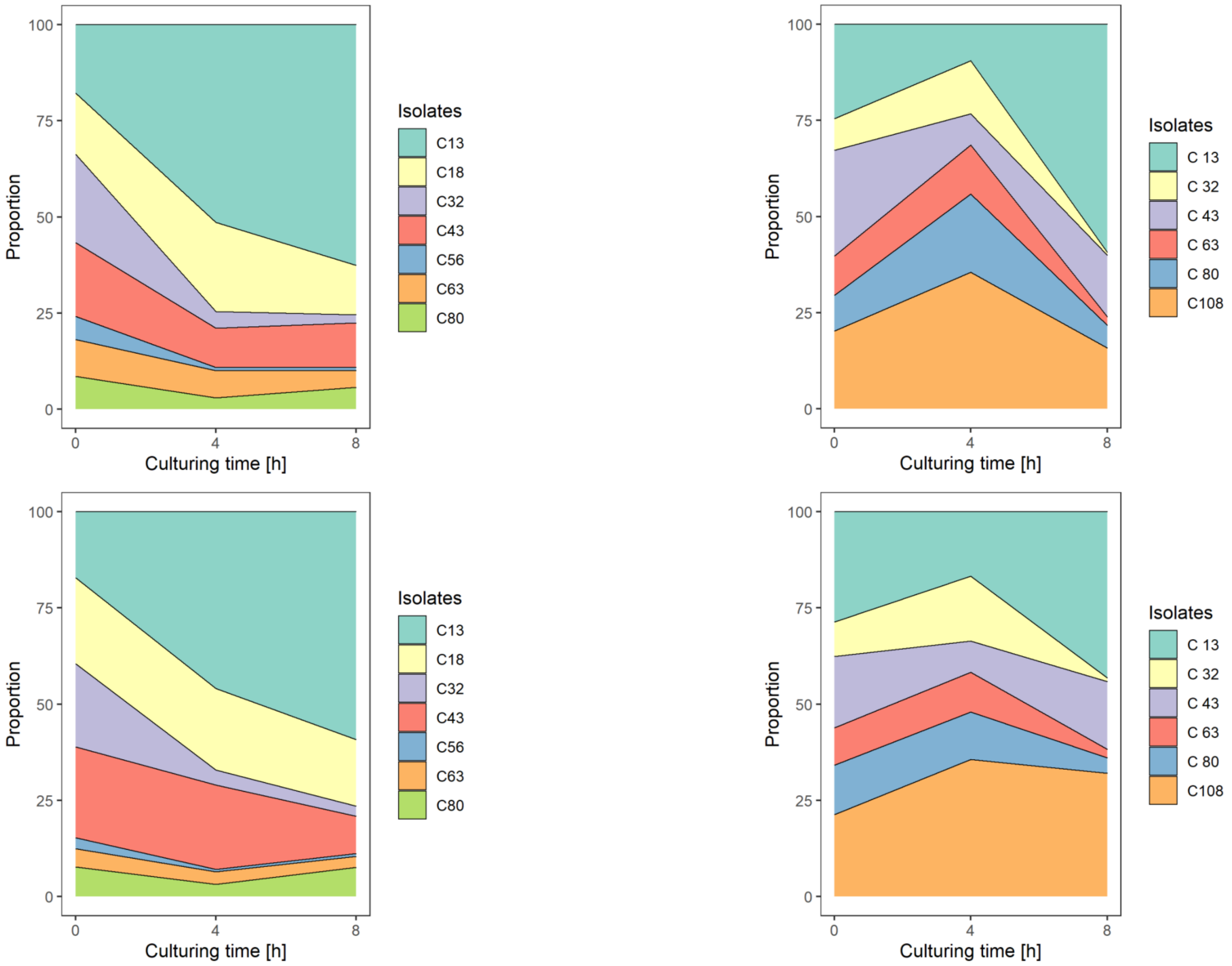

Course D

human murine

1st replicate

2nd replicate

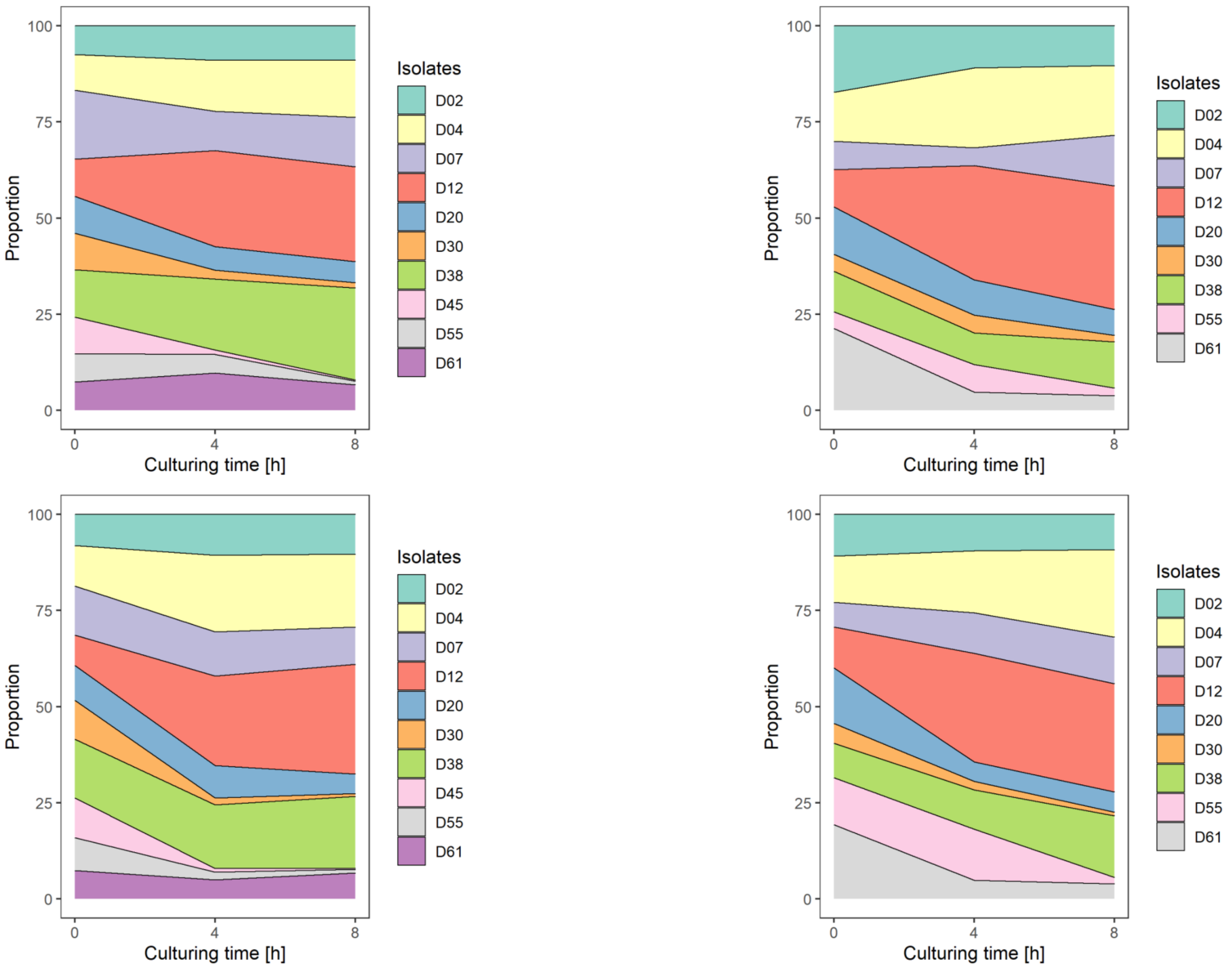

Course E

human

murine

1st replicate

2nd replicate

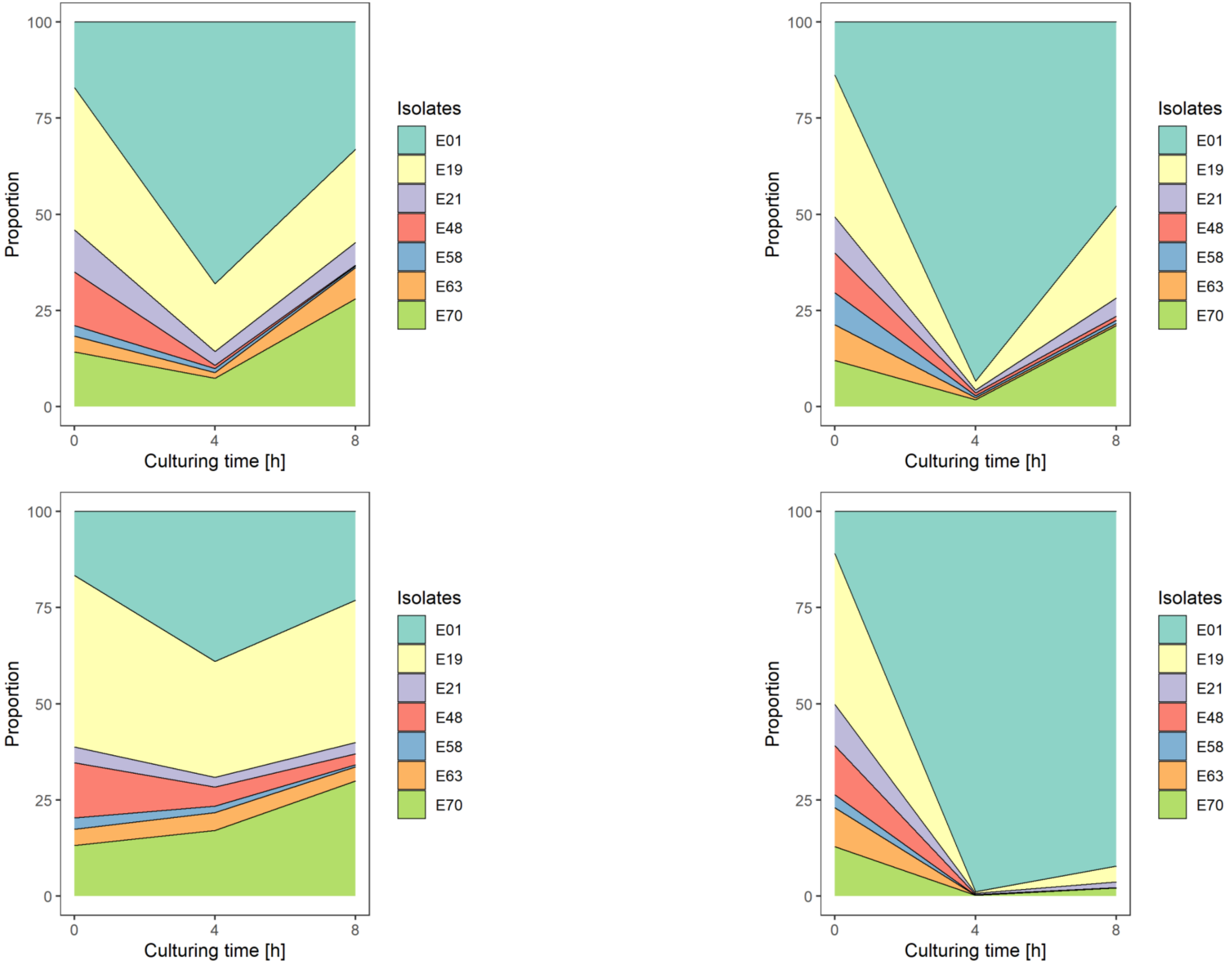

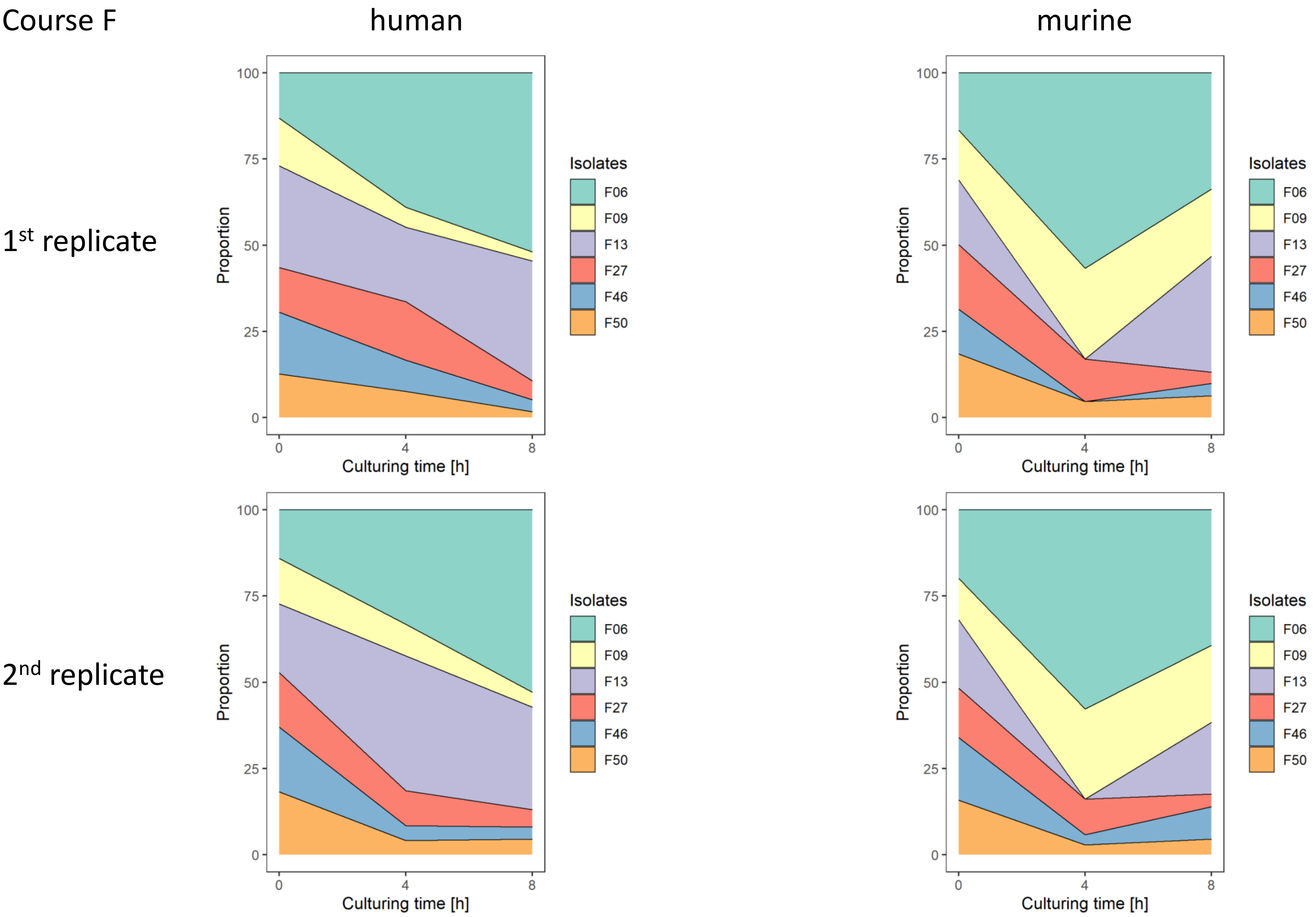

Course F
human
murine
1st replicate
2nd replicate
Proportion
100
75
50
25
0
Culturing time [h]
0
4
8
Isolates
F06
F09
F13
F27
F46
F50

Course G

human | murine

1st replicate

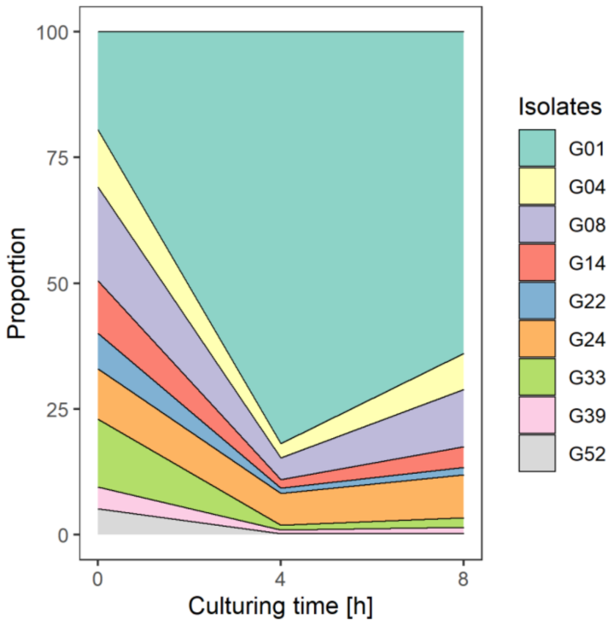

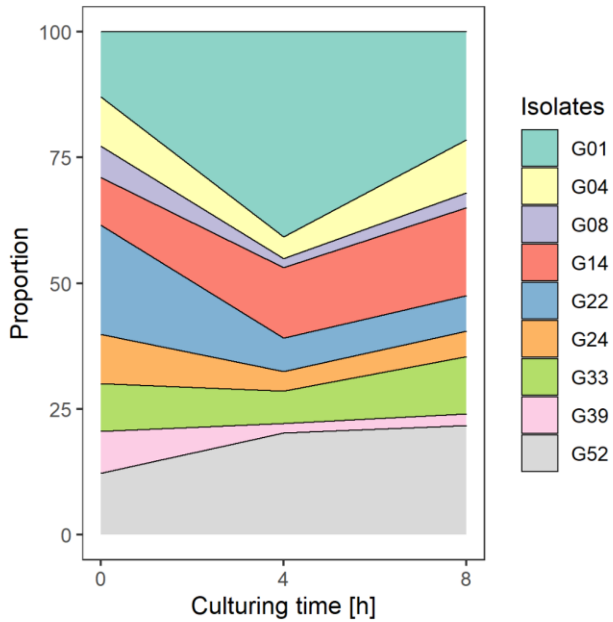

2nd replicate

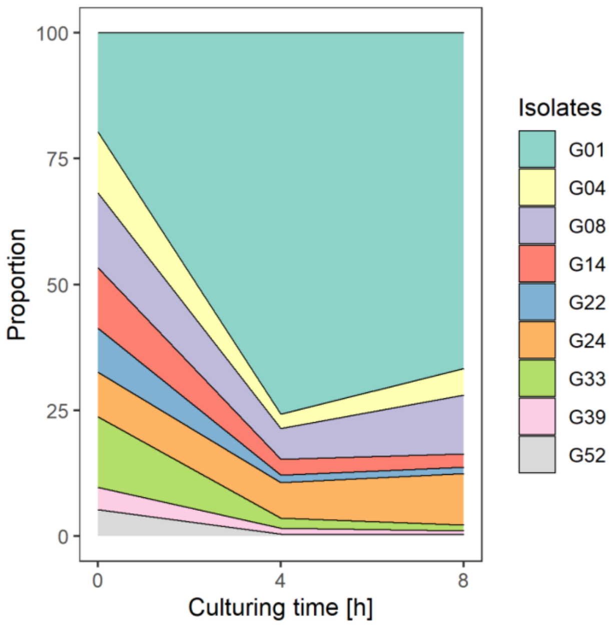

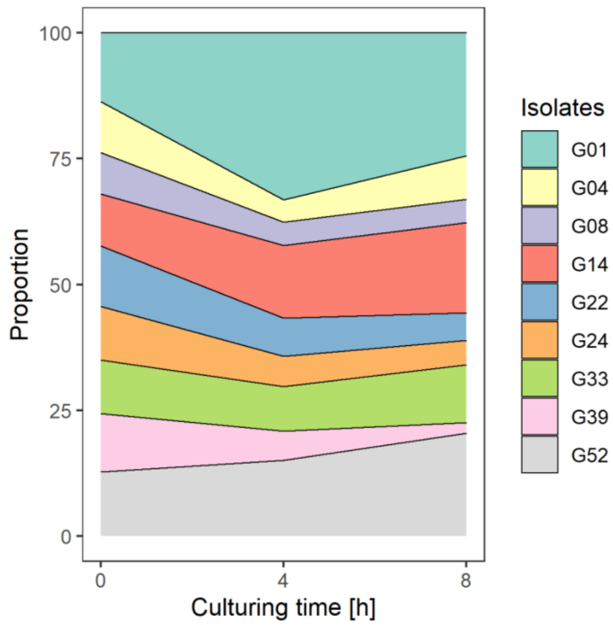

Course H

human

murine

1st replicate

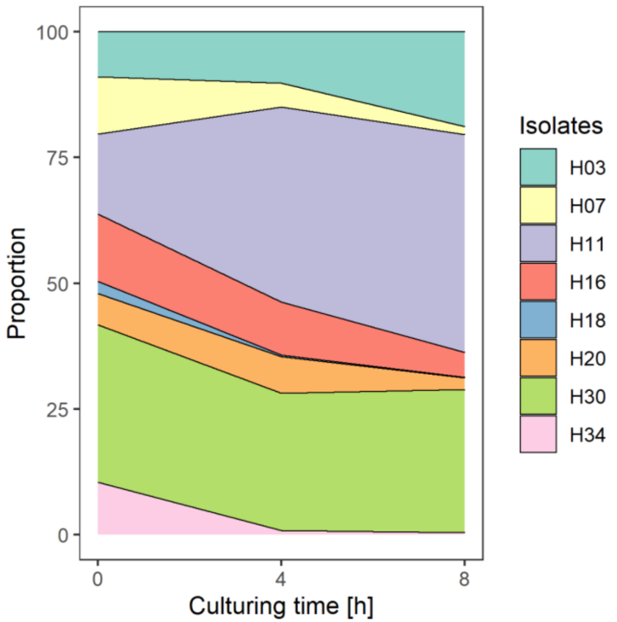

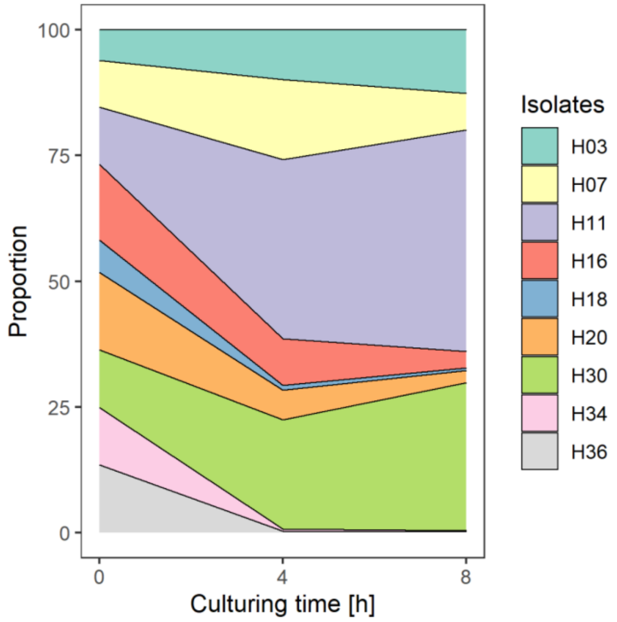

2nd replicate

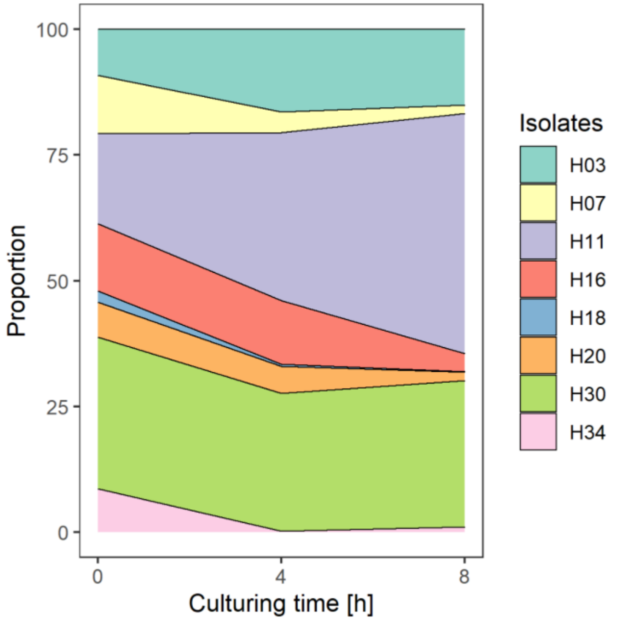

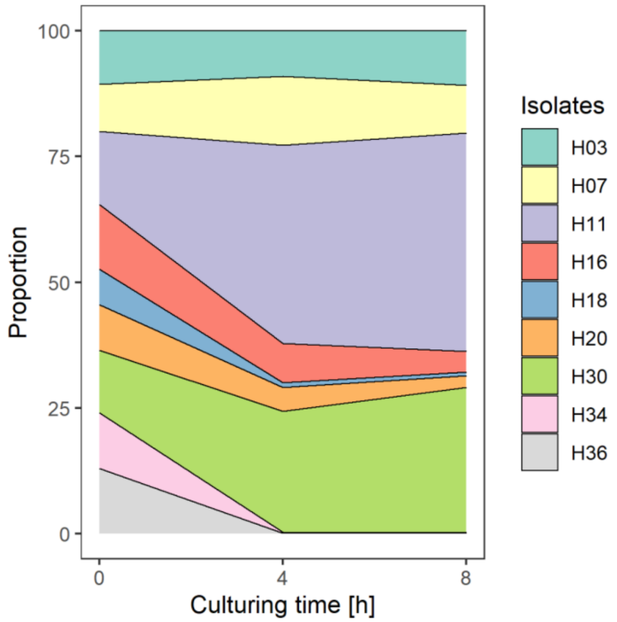

Course K

human | murine

1st replicate

2nd replicate

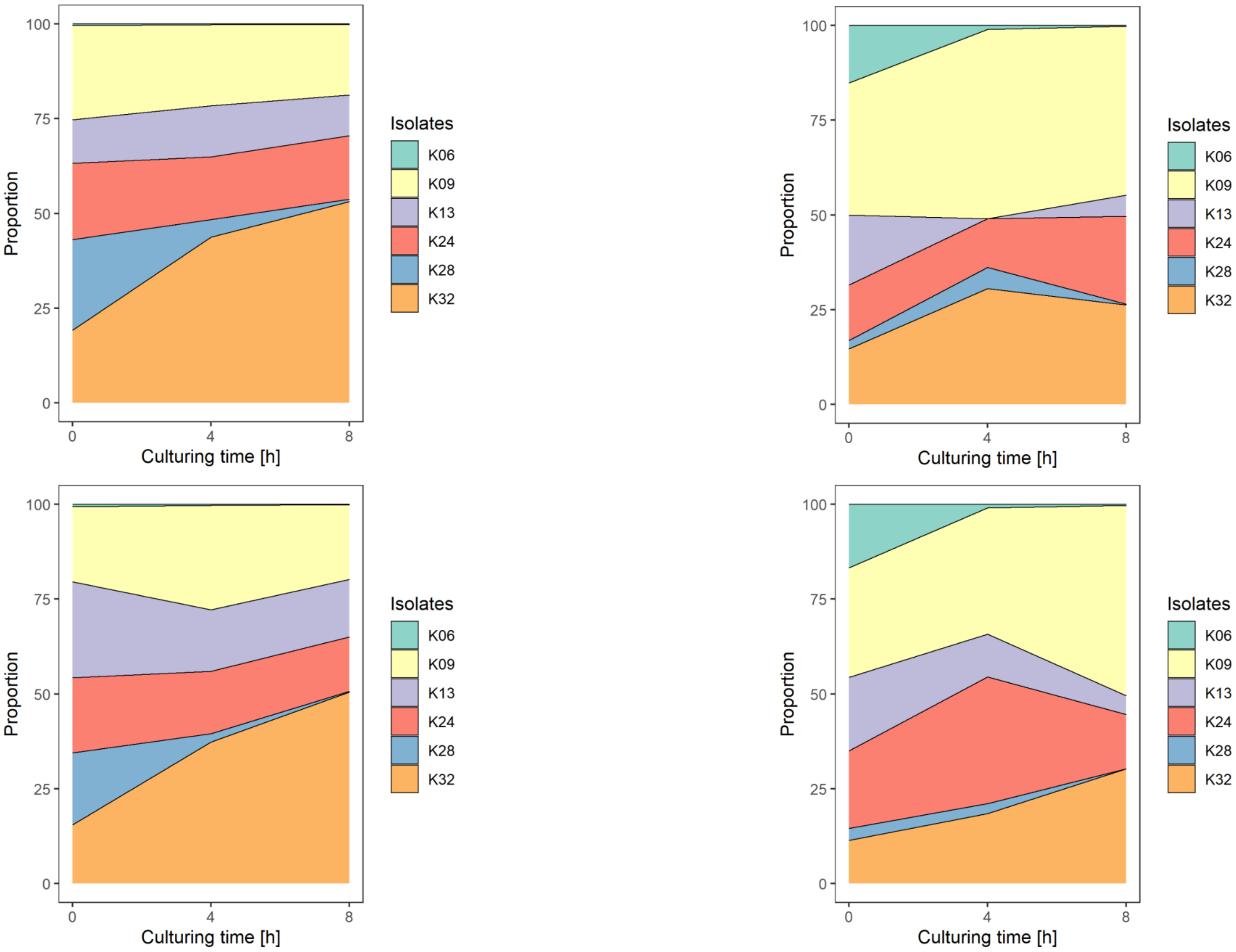

Course L

human

murine

1st replicate

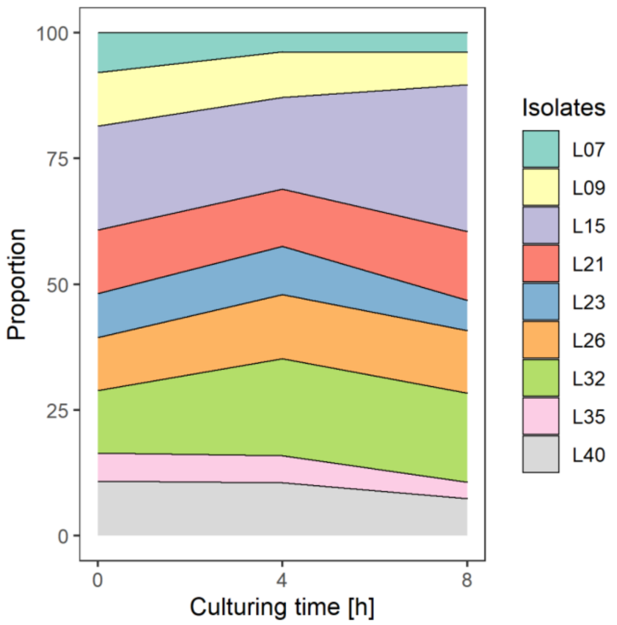

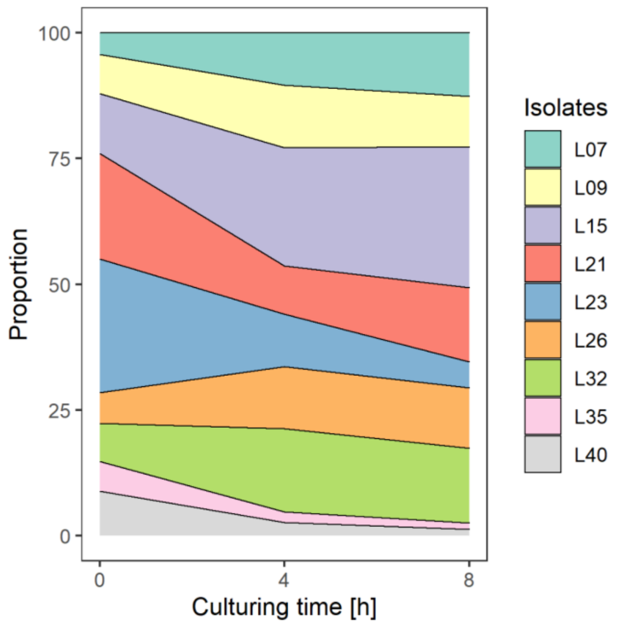

2nd replicate

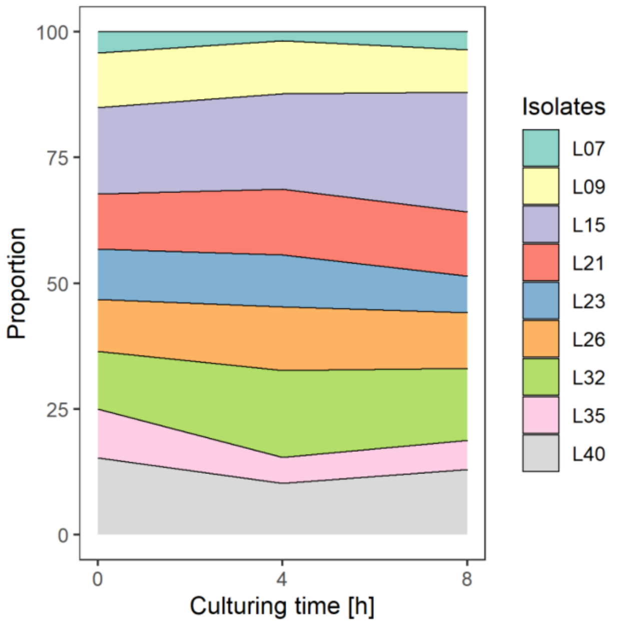

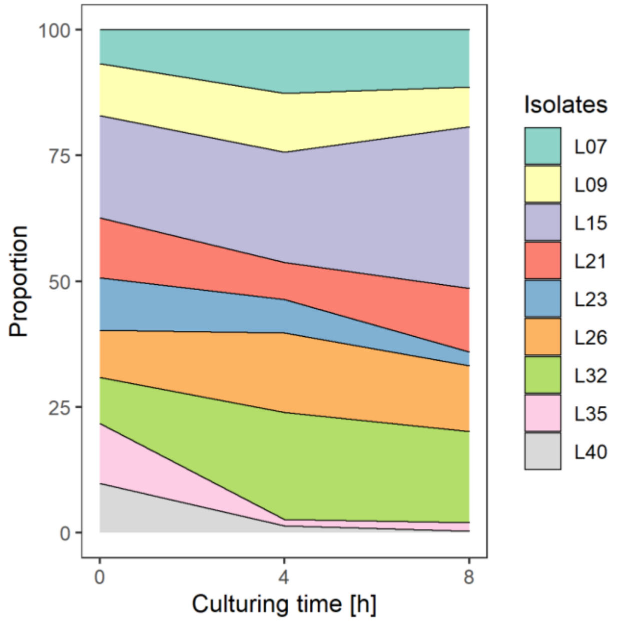

Course M

human

murine

1st replicate

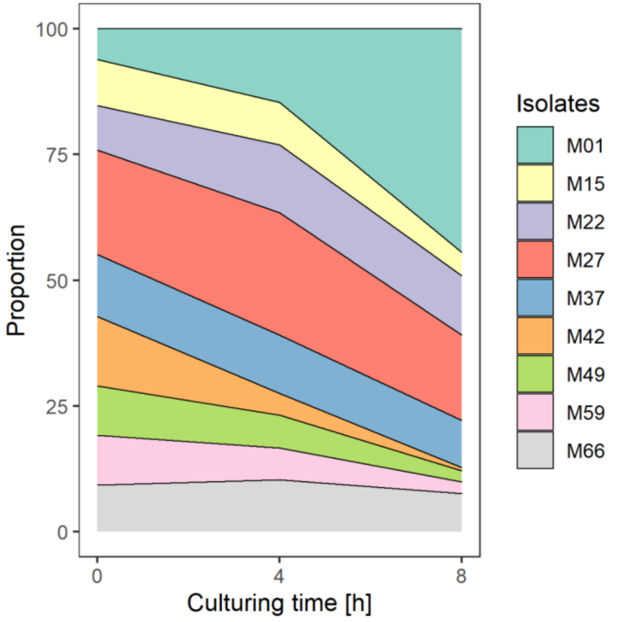

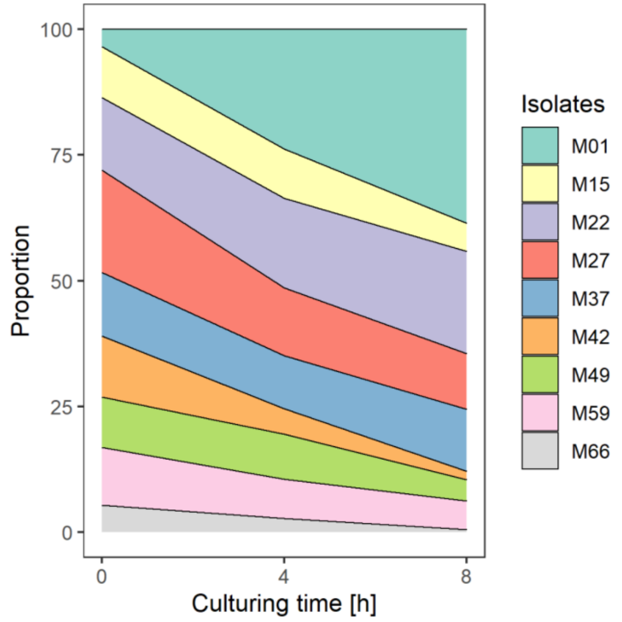

2nd replicate

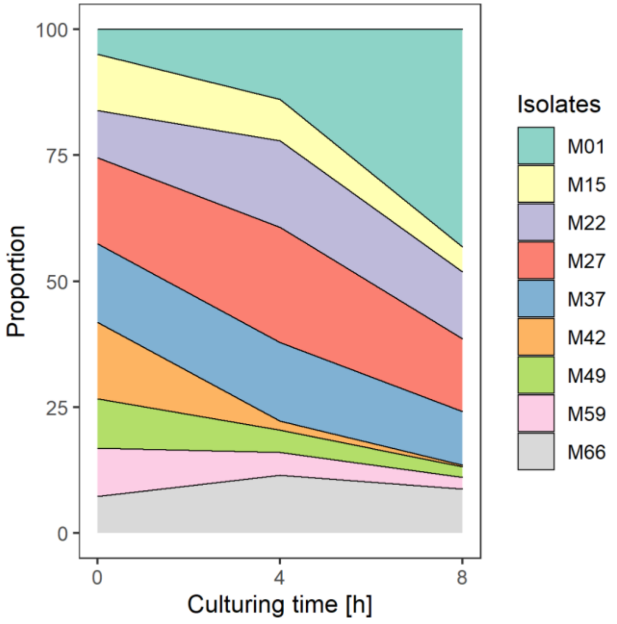

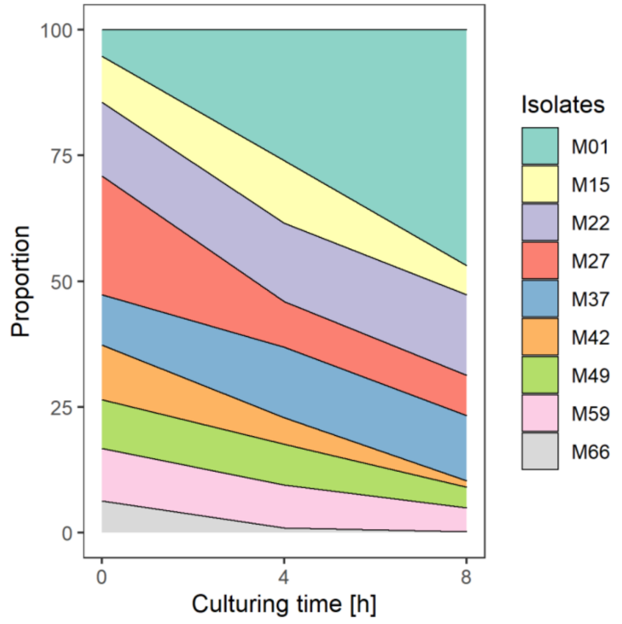

Course N

human | murine

1st replicate

2nd replicate

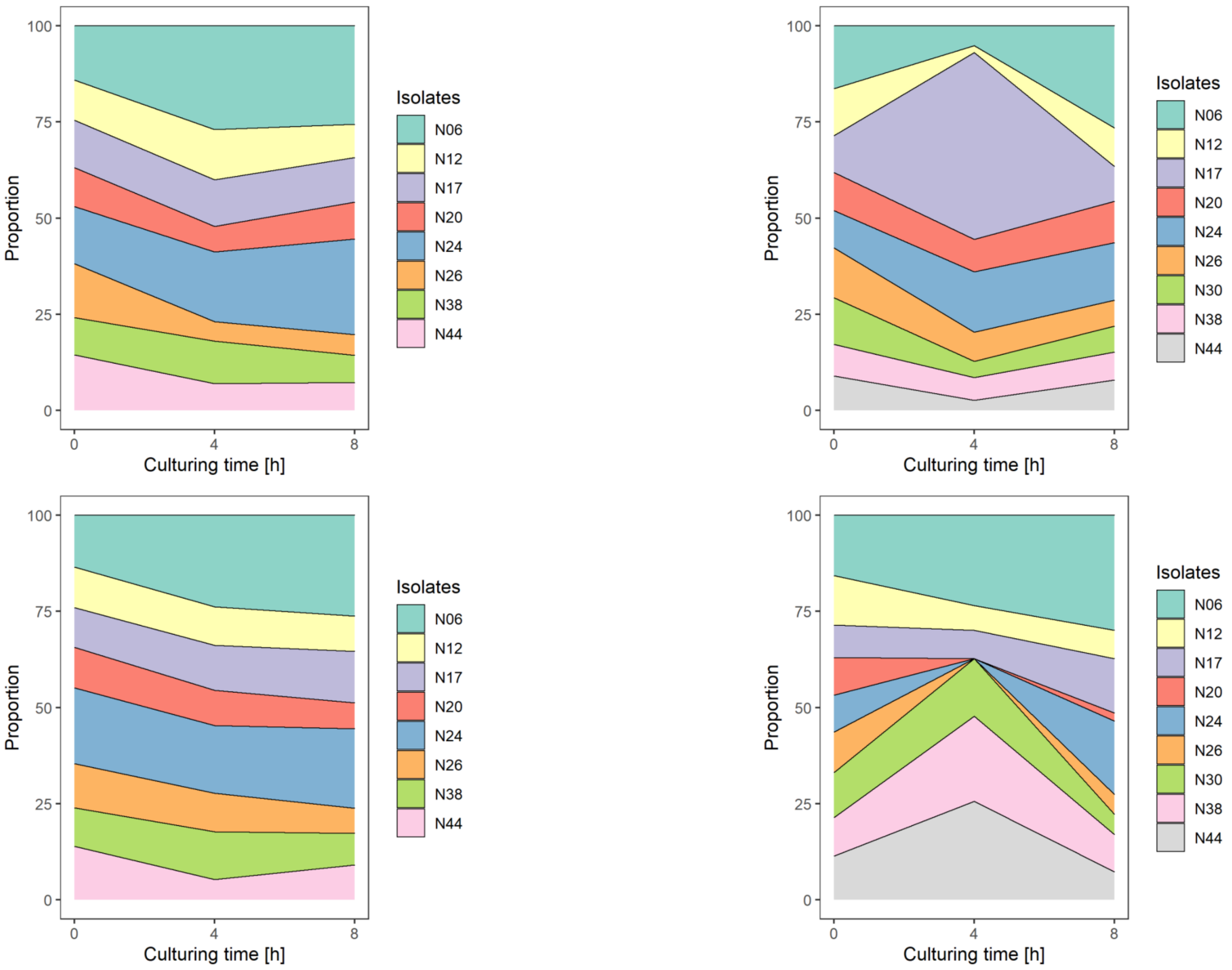

Supplement: Supplementary Data Sheet 1 — Step-by-step explanation of the evaluation of the competition experiments in murine and human PCLS. [file DataSheet_1.pdf]
